# The Intersection of Persuasive System Design and Personalization in Mobile Health: Statistical Evaluation

**DOI:** 10.2196/40576

**Published:** 2022-09-14

**Authors:** Aleise McGowan, Scott Sittig, David Bourrie, Ryan Benton, Sriram Iyengar

**Affiliations:** 1 School of Computing Sciences and Computer Engineering The University of Southern Mississippi Hattiesburg, MS United States; 2 University of Louisiana at Lafayette Lafayette, LA United States; 3 University of South Alabama Mobile, AL United States; 4 University of Arizona Pheonix, AZ United States

**Keywords:** persuasive technology, personalization, psychological characteristics, self-efficacy, health consciousness, health motivation, personality traits, mobile health, mHealth, mobile phone

## Abstract

**Background:**

Persuasive technology is an umbrella term that encompasses software (eg, mobile apps) or hardware (eg, smartwatches) designed to influence users to perform preferable behavior once or on a long-term basis. Considering the ubiquitous nature of mobile devices across all socioeconomic groups, user behavior modification thrives under the personalized care that persuasive technology can offer. However, there is no guidance for developing personalized persuasive technologies based on the psychological characteristics of users.

**Objective:**

This study examined the role that psychological characteristics play in interpreted mobile health (mHealth) screen perceived persuasiveness. In addition, this study aims to explore how users’ psychological characteristics drive the perceived persuasiveness of digital health technologies in an effort to assist developers and researchers of digital health technologies by creating more engaging solutions.

**Methods:**

An experiment was designed to evaluate how psychological characteristics (self-efficacy, health consciousness, health motivation, and the Big Five personality traits) affect the perceived persuasiveness of digital health technologies, using the persuasive system design framework. Participants (n=262) were recruited by Qualtrics International, Inc, using the web-based survey system of the XM Research Service. This experiment involved a survey-based design with a series of 25 mHealth app screens that featured the use of persuasive principles, with a focus on physical activity. Exploratory factor analysis and linear regression were used to evaluate the multifaceted needs of digital health users based on their psychological characteristics.

**Results:**

The results imply that an individual user’s psychological characteristics (self-efficacy, health consciousness, health motivation, and extraversion) affect interpreted mHealth screen perceived persuasiveness, and combinations of persuasive principles and psychological characteristics lead to greater perceived persuasiveness. The *F* test (ie, ANOVA) for model 1 was significant (*F*_9,6540_=191.806; *P*<.001), with an adjusted *R*^2^ of 0.208, indicating that the demographic variables explained 20.8% of the variance in perceived persuasiveness. Gender was a significant predictor, with women having higher perceived persuasiveness (*P*=.008) relative to men. Age was a significant predictor of perceived persuasiveness with individuals aged 40 to 59 years (*P*<.001) and ≥60 years (*P*<.001). Model 2 was significant (*F*_13,6536_=341.035; *P*<.001), with an adjusted *R*^2^ of 0.403, indicating that the demographic variables self-efficacy, health consciousness, health motivation, and extraversion together explained 40.3% of the variance in perceived persuasiveness.

**Conclusions:**

This study evaluates the role that psychological characteristics play in interpreted mHealth screen perceived persuasiveness. Findings indicate that self-efficacy, health consciousness, health motivation, extraversion, gender, age, and education significantly influence the perceived persuasiveness of digital health technologies. Moreover, this study showed that varying combinations of psychological characteristics and demographic variables affected the perceived persuasiveness of the primary persuasive technology category.

## Introduction

### Background

Given the ubiquitous nature of mobile devices across all socioeconomic groups, digital health technologies have demonstrated their efficacy as key components in educating and treating patients [1]. Mobile health (mHealth) uses mobile devices to practice medicine and public health. Unlike clinic-based treatments, where health care data are sparingly personalized, the ever-present nature of digital health technologies allows for an extensive and more intimate treatment plan. Although digital health technologies allow for the real-time transfer of user data, which allows for more intimate user interaction, these technologies are met with a unique set of challenges, such as creating and maintaining engagement [2]. The efficacy of digital health technologies relies strongly on their ability to continuously engage and re-engage users [3]. The closed-loop engagement process begins with engagement and continuously moves through disengagement to allow the patient to re-engage upon disengagement [4,5]. Properly engaging patients has repeatedly been shown to improve patient outcomes [2].

However, at the core of engagement using digital health technologies, there remains a gap in the literature on how to successfully design these tools based on an individual’s dynamic psychological makeup. For instance, there remains a need to learn more about how mHealth treatments work and how to make them more effective. In particular, research on the impact of certain intervention features on user engagement is an important next step in the development of theory and evaluation to develop a science for user engagement [6]. Although the positive influence of persuasion on changing an individual’s attitude and behavior has been established [7,8], researchers have contended the need for personalized systems that address individual’s personalities to increase the effectiveness of these tools [9,10]. One-size-fits-all digital health technologies that target behavior change to improve the user’s health often fail because they do not target the psychological traits that drive an individual’s motivations and behaviors, partly because of the lack of guidance from intervention designers and data scientists with numerous options [11]. A dynamic personalized approach to developing persuasive technologies is imperative, as research has shown that strategies that may influence change in an individual with one psychological type may dissuade another individual with a different psychological type [12].

User engagement is a widely used multifaceted term that extends beyond a user’s desire to use digital health technologies to the depth of the user’s investment [13]. Digital health technologies developers are often tasked with developing tools designed to engage patients, yet little emphasis has been placed on understanding what motivates users to engage with digital health technologies. Developers must move past using a cookie-cutter, one-size-fits-all solution, and seek to develop digital health technologies designed to traverse the fluid terrain that navigates between the expectations of the user and the technological capabilities of the tool. The fluid nature of goals and user preferences determined by user characteristics must also be considered in order to foster various engagement trajectories with digital health technologies. Synonymous with the engagement process, the development of digital health technologies must be dynamic in nature, traversing between design and redesign guided by use [14]. The unconscious disregard for the interdependency among technology, human characteristics, and the socioeconomic environment has been determined to be one of the factors in digital health technologies failing to sustain innovations in the health care field [15,16].

Persuasive technology has emerged as a significant contributor to patient engagement and is used practically in every area of health and wellness [7,17]. Persuasive technology is an umbrella term that encompasses any software (eg, mobile apps) or hardware (eg, smartwatches) designed to influence users to either perform a preferable behavior once or on a long-term basis. These modifications must be achieved without the use of deception, coercion, or inducements [18,19]. By adequately applying persuasive technology, intervention developers have the potential to improve patient outcomes by successfully closing the engagement loop. The modification of user behavior thrives under personalized care that persuasive technology must offer. However, absent from the current literature is adequate information on *how* app designers are to operationalize persuasive design principles based on a more user-centric view [20]. Research is immersed in studies related to the user experience derived from metrics and quantifications, but there remains a void in the literature seeking a more intimate view of the consumer and how they interact with persuasive principles to help guide design processes. The design process is further impaired by a lack of understanding of the psychological characteristics of digital health technology users [21]. Previous research has focused on the development of theories concentrated on predicting acceptance or adherence instead of guiding persuasive technology design principles [22]. This research is needed to fill the gap in the literature addressing the user-centric development of persuasive technologies and developing a better understanding of the psychological characteristics necessary for the successful engagement of digital health technology users.

### Consumer and Patient Engagement

There is consensus that an implicit level of engagement is required for digital health technologies to be effective. The absence of engagement impedes digital health technologies from attaining their full potential [23]. This emerging stream of research is built on a somewhat challenging and unstable foundation, as the authors used various procedures to measure engagement [24]. With various metrics in play, the ability to quantify engagement is a daunting and challenging task [24,25]. This ambiguity further exacerbates our efforts to assess effective engagement.

Digital health technology developers must exercise quantitative and qualitative methods when designing engaging applications [26]. Quantitative measures evaluating intensity and breadth of use are often used to determine the level of consumer engagement [27]. Such a holistic view is not always feasible for developers, but the use of tangible metrics (eg, the amount of screen time of the digital health technology and the number of likes and shares) can be quantified and used for quantitative data [3]. For engagement to be meaningful, digital health technologies must modify user behavior and advance ordinary experiences into aesthetically pleasing ones [28].

Chapman et al [29] proposed that engagement was dichotomous, being either less passive or more passive based on the level of control. More controlled engagement requires information processing such as critical thinking and reasoning and involves a less passive state of engagement. Passive engagement requires less control and is easier to achieve, because the level of effort and motivation is low. Although easier to achieve and maintain, passive engagement is less useful in the successful achievement of established goals that require high levels of cognition [29].

The delivery of appropriately tailored digital health technology content can increase users’ engagement and positively influence outcomes. This makes it imperative to understand how to design digital health technologies based on patient and consumer preferences [30]. Identifying the features of digital health technologies that stimulate user engagement is crucial for developing effective tools [21]. One of the key factors in the development of digital health technologies that enhance engagement through the aforementioned techniques is persuasive technology.

Characteristics such as gender, age, and personality affect how users respond to persuasive technologies, causing a pivot from one-size-fits-all solutions to a more user-centric approach [12]. Persuasive technologies can adapt to the individualized characteristics of users, increasing their likelihood of changing their behavior or attitude [31]. Studies show that persuasive technologies that personalize content instead of using one-size-fits-all approaches are more successful in effectively persuading users [32-34]. One-size-fits-all persuasive technologies can be enhanced when a user’s individual attitudes and characteristics are used to influence and personalize the persuasiveness of the intervention [35].

Although research has shown that individualized persuasive technology is more effective than persuasive technology designed from a one-size-fits-all perspective [36-38], developers often fail to consider the individualized behavior of stakeholders and how it impacts achieving a target behavior [39]. Digital health technologies that deviate from compartmentalized one-size-fits-all approaches offer a medium through which health care providers can meet the growing demands of users, preferring a more personalized approach [26,40]. This growing demand necessitates the ability to understand how to design digital health technologies that are dynamic enough to accommodate the differing predispositions of end users [30].

Designers must understand how to tailor digital health technologies according to individual characteristics to effectively engage users with these tools. By tailoring digital health technologies to users’ characteristics, developers can deliver guidance that is appropriate, relevant, and has a positive impact on engagement [41]. Disregard for the interconnectedness between human characteristics and technology is one reason digital health technologies inevitably become high technology with little to no impact [42]. Current theories are inept at informing digital health technology developers on how to develop and evaluate more adaptive interventions [43,44]. Recognizing the psychological characteristics of end users will allow developers to systematically approach the integration of persuasive design components into digital health technologies.

Data-centered persuasive technologies seek to modify user attitudes or behaviors through users’ behavioral data [45]. Current technology allows intervention designers to dynamically generate personalized interventions based on a specific user’s personal characteristics [46]. Dynamic approaches acknowledge that interventions designed for one user may not necessarily fit the model required to effectively engage another user. User characteristics often dictate the most effective persuasive technique [35]. Persuasive technologies applicable to the health care domain are more effective when personalized based on the user’s personal characteristics [47]. Because personalized persuasive techniques evoke a different response from more traditional, one-size-fits-all techniques, intervention designers must shift to a more individualized approach guided by the individual’s preferences [12].

Personalized interventions that target nuances that drive users’ choices and behaviors are better suited to facilitate effective engagement than black box, one-size-fits-all solutions [11]. It has long been established that personalized content is more effective as it increases user attention, leading to effective engagement [32]. The application of data collected from individuals is a more advanced method of persuasion that increases the probability of success and results in more active and effective intervention [45]. Determining the key data elements to collect to enhance perceived persuasiveness is critical in efforts to improve engagement (both in the short and long term).

### Psychological Characteristics

#### Self-efficacy

Self-efficacy is loosely defined as an individual’s belief that they are capable of successfully executing courses of action required to successfully produce specific behaviors [48]. An individual’s estimate of self-efficacy varies in 3 dimensions: magnitude (the individual’s belief in their ability to complete a task), strength (the individual’s confidence that they are capable of completing various components or varying levels of difficulty in a task), and generality (the extent to which an individual’s self-efficacy transfers from one task to related tasks) [48,49]. Self-efficacy is regarded as a core premise of human performance, as demonstrated by its use across multiple domains including education [50,51], exercise [52], physical activity [53], career [54], and health care [55].

Individuals avoid tasks that they presume to exceed their ability levels [56]. Situations in which these tasks occur affect an individual’s evaluation of self-efficacy. Self-efficacy is more likely to increase when individuals are able to ascribe success, as opposed to failure, to their individual skill set [56,57]. The difficulty level of a task also correlates with an individual’s appraisal of self-efficacy [58]. Tasks deemed difficult to successfully complete tend to have a negative effect on an individual’s appraisal of self-efficacy [59]. According to Bandura [56], individuals will go so far as to be unwilling to attempt to manage situations where their low self-efficacy indicates a negative outcome [60].

Hypothesis 1: self-efficacy will positively influence interpreted mHealth screen perceived persuasiveness.

#### Health Consciousness

Health consciousness is defined as the measure to which an individual integrates health concerns into their daily regime [61-63]. Unlike health motivation (HM), which is external in nature, health consciousness refers to “how” an individual achieves a healthy lifestyle [61]. Research has shown that the higher an individual’s health consciousness, the more likely they are to adopt a lifestyle grounded in health behaviors such as fitness and nutritional activities [62,64]. These individuals are cognizant of their health and therefore influenced to adopt these healthier behaviors needed to improve or maintain their health [65].

Studies have shown that health consciousness can positively influence engagement in health-oriented actions [66]. This motivation to engage in health-oriented actions has the propensity to push individuals to become connoisseurs of health information via media sources such as television [64] and the internet [67]. Also observed has been the correlation between the increase in health consciousness and the increase in preventive health care [61,68]. Individuals with high health consciousness reportedly seek to develop and preserve a healthy lifestyle [69].

Hypothesis 2: health consciousness will positively influence interpreted mHealth screen perceived persuasiveness.

#### Health Motivation

HM is closely related to health consciousness, as it is one of the 3 elements that comprise health consciousness [67]. HM is an individual’s drive to engage in health-related activities to improve or maintain preventive health behaviors [61,70]. HM has been found to be a relatively consistent state deeply rooted in an individual’s psychological composition [61]. Research has shown that HM serves as the source of an individual’s desire, adoption, and practice of preventive health behaviors [61,70]. Motivation has been found to be both competency-based (whether a person can achieve the goal) and goal-oriented (the way a task is managed is determined by the individual’s objective) [71].

It has also been determined that HM can gauge an individual’s well-being with regard to health behavior–related concerns and actions [72] and drive consumer engagement in health maintenance behaviors [70]. HM is directly linked to an individual’s internal characteristics [61]. Research has consistently shown that internalized motivation results in more pronounced adherence to preventive health behaviors such as weight loss [73,74]. Whether an individual expects to succeed also plays a key role in their degree of motivation [75].

Hypothesis 3: HM will positively influence interpreted mHealth screen perceived persuasiveness.

#### Personality Traits

Personality traits and strategies used to engage users have an impact on the effective engagement of digital health technology [76]. Understanding these personality traits is critical for creating digital health solutions that meet the needs of users. One of the most commonly used personality models is the Big Five factor model [77]. The Big Five factor framework was developed by Goldberg [78] and later validated by Costa and McCrae [78,79]. This model delineated five factors of personality:

1. Openness to experience: the extent to which an individual requires intellectual stimulation, change, and variety
2. Conscientiousness: the extent to which an individual is willing to comply with conventional rules, norms, and standards
3. Extraversion: the extent to which an individual needs attention and social interaction
4. Agreeableness: the extent to which an individual needs pleasant and harmonious relationships with others
5. Neuroticism: the extent to which an individual observes the world as threatening and beyond their control [80]

**Figure 1:**
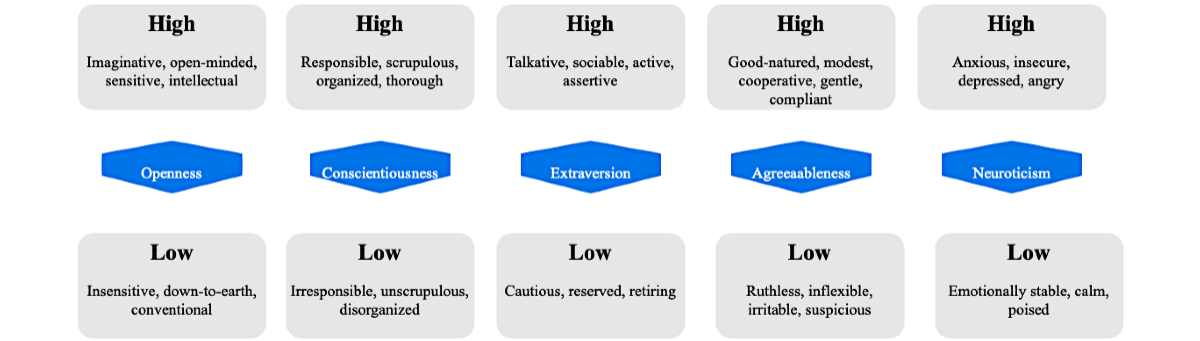
Big Five continuum.

Each Big Five personality category can be regarded as a continuum in which individual scores range from high to low (Figure 1 [77]).

Frequent time constraints are the drivers for a more succinct measurement tool [81]. The Mini-International Personality Item Pool (Mini-IPIP) Scale is a condensed 20-item diagnostic tool that has been validated in multiple studies [82]. Researchers have used the Big Five framework to predict user characteristics across a conglomerate of domains: career [83], relationship satisfaction and love styles [84], academic performance [85], preventive health care [86], and more.

Individual personality traits often reflect not only what drives and motivates people but also what they prefer. The Big Five personality dimensions describe human behavior in 5 dimensions: openness, conscientiousness, extraversion or introversion, agreeableness or disagreeableness, and neuroticism. Individual personality traits should be an antecedent of consumer engagement with mHealth apps [81].

Hypothesis 4: openness will positively influence interpreted mHealth screen perceived persuasiveness.

Hypothesis 5: conscientiousness will positively influence interpreted mHealth screen perceived persuasiveness.

Hypothesis 6: extraversion will positively influence interpreted mHealth screen perceived persuasiveness.

Hypothesis 7: agreeableness will positively influence interpreted mHealth screen perceived persuasiveness.

Hypothesis 8: neuroticism will negatively influence interpreted mHealth screen perceived persuasiveness.

## Methods

### Ethics Approval

Institutional review board approval was obtained from the University of South Alabama (application 18-353/1314060-1).

### Overview

To examine the factors related to engagement behavior with the intention to use an mHealth app, a multiple-phase experiment was conducted in the summer of 2020. This experiment involved a survey-based design with a series of 25 mHealth app screens that featured the use of persuasive principles, with a focus on physical activity. This study used exploratory factor analysis (EFA) and multiple linear regression to aid designers in the user-centric development of persuasive technologies. This study aimed to develop a better understanding of the psychological characteristics necessary for the successful engagement of digital health technology users.

### Recruitment

Participants were recruited by Qualtrics International, Inc to use the web-based survey system by XM Research Service [87], which has been previously used by researchers in a variety of disciplines [88,89]. Qualtrics reimbursed participants with a predetermined amount of money arranged between Qualtrics and participants. Once interested participants were selected by Qualtrics, they were directed to the informed consent page via an anonymous link. Upon consenting to participate, they were directed to a web-based engagement screen survey. Participants were recruited between July 23, 2020, and August 3, 2020. The engagement screen survey took an average of 28.08 (19.35 SD) minutes to complete. There were 273 completed survey responses; however, 11 (4%) were deleted owing to evident signs of respondents being “speeders” that completed the survey in an impossibly quick time or “straight lining” and giving identical answer choices repeatedly, leaving this study with 262 (95.9%) viable responses.

### Screen Development

To examine the perceived persuasiveness of mHealth screens, 25 unique mHealth screens were developed following the persuasive system design (PSD) categories and principles developed by Oinas-Kukkonen and Harjumaa [90]. The screens were all developed with the central theme of improving or increasing exercise as a use case.

The mHealth screen development process began with the creation of a wireframe prototype [91]. The prototype was created on sheets of paper, with each sheet representing one of the mHealth app screens. The initial step for each prototype was to document the persuasive system category, design principle, and targeted implementation as per Oinas-Kukkonen [90]. A brief description of the details of the screen was then added to the prototype, followed by the mHealth screen being given a reference name based on the details in the write-up used throughout the questionnaire development and analysis process. Table 1 presents examples of the initial steps. A sketch of the prototype was then drawn based on the documentation so that each sheet would represent one of the mHealth screens.

BuildFire [92] was used to develop a digital high-fidelity prototype for each mobile app screen. These prototypes were used to support the design goals established in the initial prototype. Once the prototypes were developed, iPhone XS Max was used to create still images of the mHealth screens using the screenshot function. This method was used so that the image would visually represent what a user would see on their smartphone. The images were exported from the mobile phone to a laptop computer via email. Once the prototypes were exported, 2 experts in the field of persuasive technology conducted a blind review to validate the mHealth screen, representing the persuasive technology principle intended by the author. The expert review panel consisted of a reviewer with 12 years of experience in the field of persuasive technology and a reviewer with 9 years of experience in the field of persuasive technology. Each expert created a datasheet with the associated screen names and listed the PSD principles identified on each screen.

Following the expert inspection and blind review, a consultation was held with the expert review panel, where notes and suggestions were reviewed. The review and modification processes continued until the developer and reviewers reached a consensus. The mHealth screens were iteratively evaluated, modified, and improved following each expert inspection and blind review. For the initial round, 23 mHealth screens were developed: Add, Start, Burpee-Squat, Increase, Mountain, Target, Trophy, Late, Calories, Dinner Chat, Tracker, About Us, Stories, Leaderboard, Journal, Partners, Ads, Strategy, CDC, HIPAA, Contact, Before After, and Yoga. During the initial expert review, the developer and reviewers identified 11 mHealth screens with conflicting persuasive technology principles that required modification: Target, Dinner Chat, About Us, Journal, Partners, Strategy, HIPAA, Contact, Before After, Yoga, and CDC. CDC was dropped following the initial review because the designed persuasive category was not seen by either of the 2 reviewers, and the category that was identified was seen on another screen. The Apple mHealth screen was created to replace the CDC and submitted with revisions for round 2. Consensus was reached on the 23 mHealth screens during the second round. In addition, 3 paper and high-fidelity prototypes were created for the remaining mHealth screens (SSL, Avatar, and Recreation) following the aforementioned methods. Additional mHealth screens were iteratively evaluated, modified, and improved using expert inspection and blind review methods used during rounds 1 and 2. The iterative process resulted in 25 mHealth screens designed for the questionnaire that were agreed upon through the blind review process, and an mHealth screen prototype was discarded. The acceptance of the mHealth screen by round is presented in Table 2.

**Table 1:** Examples of the initial prototype development steps.

Persuasive system category and design principle			Targeted implementation		Mock-up		Mock-up name
**Primary task support**							
	Reduction	Provide simple steps for an activity		Show literature such as weight loss made simple, which gives simple steps to get started for losing weight		Start	
	Tunneling	Guiding people in a process step by step to meet a goal		Fitness program with step-by-step workout plan. Once daily or weekly goals are reached, the next set of steps are given		Burpee-Squat	
	Tailoring	The system uses factors relevant to the individual to motivate the users based on their needs, interests, personality, and so on		Users can modify the app to reflect their interests and personality (change color pallet, select what is displayed on home screen, etc)		Add	
	Personalization	Suggestions, praise, and rewards are given at appropriate time to motivate users to stay on track		Increase the user’s activity goal based on accomplishments or modify dietary plan based on weight loss		Increase	
	Self-monitoring	Allows users to follow or monitor their performance to ensure that they are staying on track		Summary of daily or weekly activity calculations and weekly weight summaries		Tracker	
**System credibility support**							
	Trustworthiness	Apps should appear to be truthful, fair, and unbiased		Display information guaranteeing HIPAA^a^ compliance to reassure users that information will not be shared with third-party organizations		HIPAA	
	Expertise	Provide content from experts (physicians or specialists)		Chat screen showing interaction with person that resembles a physician or medical professional		About Us	

^a^HIPAA: Health Insurance Portability and Accountability Act.

**Table 2:** Mobile health (mHealth) screen acceptance by round.

Screen name	Round 1	Round 2	Round 3
Add	✓^a^		
Start	✓		
Burpee-Squat	✓		
Increase	✓		
Mountain	✓		
Target		✓	
Trophy	✓		
Late	✓		
Calories	✓		
Dinner Chat		✓	
Tracker	✓		
About Us		✓	
Stories	✓		
Leaderboard	✓		
Journal		✓	
Partners		✓	
Ads	✓		
Strategy		✓	
CDC^b^	Dropped	N/A^c^	N/A
HIPAA^d^		✓	
Contact		✓	
Before After		✓	
Yoga		✓	
Apple	N/A	Replaced CDC	
SSL	N/A	N/A	✓
Avatar	N/A	N/A	✓
Recreation	N/A	N/A	✓

^a^✓: indicates that the mHealth screen was accepted.

^b^CDC: Centers for Disease Control and Prevention.

^c^N/A: not applicable.

^d^HIPAA: Health Insurance Portability and Accountability Act.

The primary task support category aids the user in performing fundamental tasks by reducing complex tasks into simpler tasks. The primary task principles include reduction, tunneling, tailoring, personalization, self-monitoring, simulation, and rehearsal [90]. Textbox 1 describes the primary task support design principles [90].

The dialogue support category facilitates human-to-computer dialogue between the persuasive system and user. The principles used to provide feedback are praise, rewards, reminders, suggestions, similarities, liking, and social roles [90]. Textbox 2 describes the principles of the dialogue support category [90].

The system credibility category represents how systems can be made more persuasive by making them more credible. The principles used to give credibility include trustworthiness, expertise, surface credibility, real-world feel, authority, third-party endorsements, and verifiability [90]. Textbox 3 describes the principles of the system credibility category [90].

Principles in the social support category motivate systems through social influence. Design principles in this category include social facilitation, social comparison, normative influence, social learning, cooperation, competition, and recognition. Textbox 4 shows the principles of social support [90].

**Figure 2:**
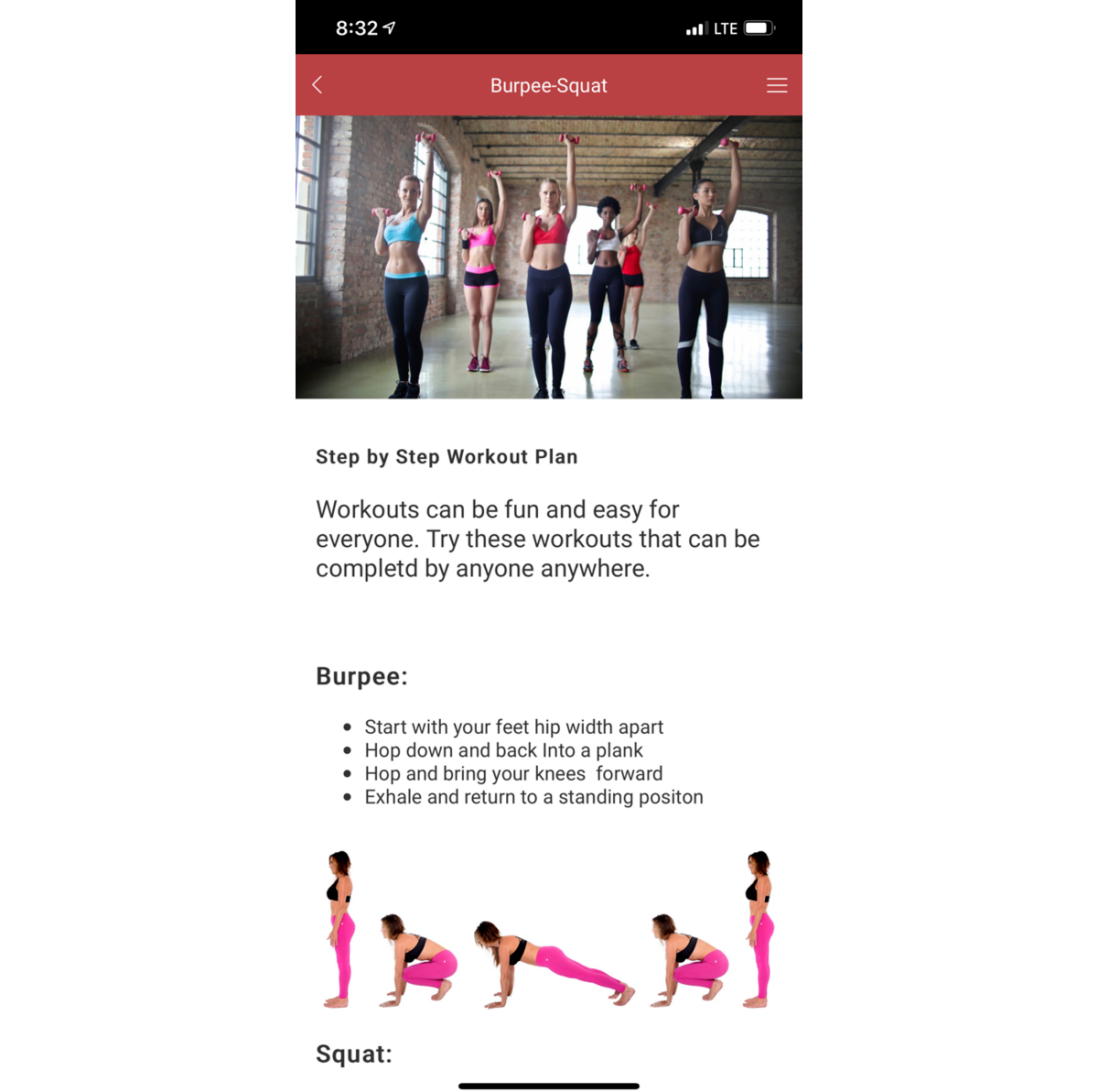
Sample mobile health screen developed and accepted during review.

Table 3 depicts the final iteration of testing and includes the principles per screen and the principle category. Table 4 shows the percentage of screens in the primary persuasive technology category. Figure 2 shows one of the final mHealth screens developed. A visual representation of all 25 screens is available in Multimedia Appendix 1. Of the 25 screens developed, 5 (20%) screens had a primary principle from the primary task support category, 7 (28%) had a primary principle from the dialogue support category, 8 (32%) had a primary principle from the system credibility support category, and 5 (20%) had a primary principle from the social support category.

**Primary task principles.**

**Persuasive system category, design principle, and principle description—primary task support**

- Reduction
  Provides simple steps for an activity
- Tunneling
  Guides people in a process step by step to meet a goal
- Tailoring
  Uses factors relevant to the individual to motivate the users based on their needs, interests, personality, and so on
- Personalization
  Suggestions, praise, and rewards are given at appropriate time to motivate users to stay on track
- Self-monitoring
  Allows users to follow or monitor their performance to ensure they are staying on track
- Simulation
  Allows the user to observe the cause-and-effect link regarding their behavior
- Rehearsal
  Allows users to rehearse a behavior

**Dialogue support principles.**

**Persuasive system category, design principle, and principle description—dialogue support**

- Praise
  Uses images, words, sounds, and so on to praise the user for their behavior
- Rewards
  Uses web-based rewards, given to the user for performing tasks related to the target behavior
- Reminders
  Reminds the user of their target behavior
- Suggestion
  Offers the user suggestions that fit the target behavior
- Similarity
  Remind users of themselves in some way
- Liking
  The digital health technology should be visually attractive
- Social role
  The digital health technology adopts a social role

**System credibility principles.**

**Persuasive system category, design principle, and principle description—system credibility support**

- Trustworthiness
  Apps should appear to be truthful, fair, and unbiased
- Expertise
  Provide content from sources that are knowledgeable and competent
- Surface credibility
  Systems should visually appear to be competent and credible
- Real-world feel
  Systems should highlight the people or organizations that are providing content by providing information about them
- Authority
  Systems should leverage roles of authority by referring to organizations and people that are seen as authority figures
- Third-party endorsements
  Systems should provide users with endorsements from third parties that are well known and trusted
- Verifiability
  Systems should provide ways for users to easily use external sources to verify the accuracy of the content

**Social support principles.**

**Persuasive system category, design principle, and principle description—social support**

- Social learning
  The digital health technology should target behavior by providing the user with a way to observe other users who are performing the same target behavior
- Social comparison
  The digital health technology should motivate the user by allowing them to compare their performance with other users who are performing the same task
- Normative influence
  The digital health technology should use normative influence or peer pressure
- Social facilitation
  The digital health technology should allow users to perceive that other users are using the system to perform the target behavior along with them
- Cooperation
  The digital health technology should leverage the users’ natural drive to cooperate
- Competition
  The digital health technology should leverage the users’ natural drive to compete with other users
- Recognition
  The digital health technology should offer users public recognition

**Table 3:** Mobile app screen name with persuasive principles and categories.

Screen name	Principle 1 (primary)	Principle 2	Principle 3
Add	PT^a^: tailoring	PT: tunneling	—^b^
Start	PT: reduction	PT: tunneling	—
Burpee-Squat	PT: tunneling	PT: reduction	—
Increase	DS^c^: praise	—	—
Mountain	PT: rehearsal	DS: suggestion	—
Target	DS: praise	PT: personalization	—
Trophy	DS: rewards	DS: praise	—
Late	DS: reminders	—	—
Calories	DS: suggestion	—	—
Dinner Chat	DS: social role	DS: praise	—
Tracker	PT: self-monitoring	—	—
About Us	SC^d^: expertise	SC: trustworthiness	SC: authority
Stories	SS^e^: recognition	PT: simulation	DS: praise
Leaderboard	SS: competition	—	—
Journal	SS: social learning	SS: social comparison	SC: social facilitation
Partners	SC: trustworthiness	SC: expertise	SC: authority
Ads	SC: surface credibility	—	—
Strategy	SC: authority	SC: expertise	—
Apple	SC: verifiability	SC: expertise	SC: authority
HIPAA^f^	SC: trustworthiness	SC: surface credibility	—
Contact	SC: real-world feel	—	—
Before After	SC: normative influence	PT: simulation	—
Yoga	SS: cooperation	DS: praise	SS: social comparison
SSL	SC: third-party endorsements	SC: trustworthiness	—
Avatar	DS: similarity	DS: liking	—

^a^PT: primary task support.

^b^Not available.

^c^DS: dialogue support.

^d^SC: system credibility support.

^e^SS: social support.

^f^HIPAA: Health Insurance Portability and Accountability Act.

**Table 4:** Screen category breakdown.

Persuasive technology category	Mobile screens (%)
Primary task support	20
Dialogue support	28
System credibility support	32
Social support	20

**Figure 5:**
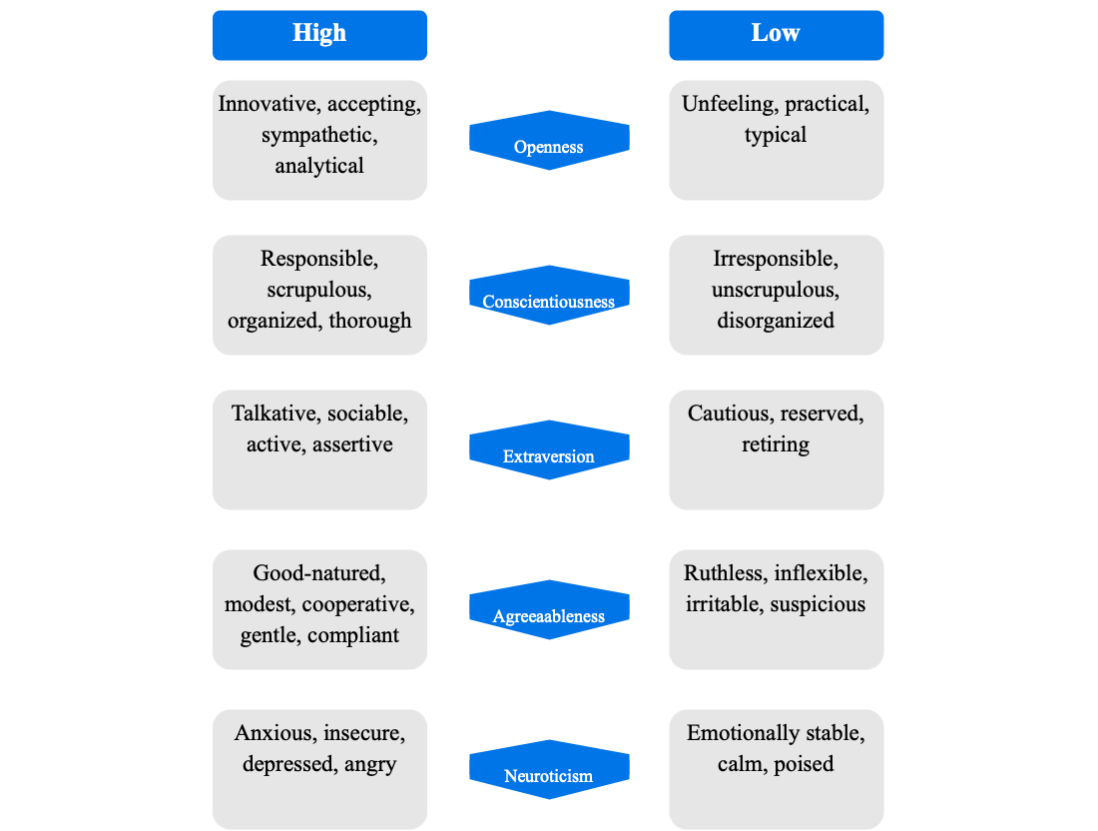
Big Five continuum.

### Measurement Items

#### New General Self-Efficacy Scale

After completion of the social demographic information, the participants were asked 8 questions about their self-efficacy using the New General Self-Efficacy Scale (Multimedia Appendix 2) by Chen et al [93]. The work by Chen et al [93] extends the work by Bandura [48,56], which focuses on the magnitude and strength dimensions of self-efficacy and includes the generality dimension of self-efficacy. Data on self-efficacy were collected from participants at baseline and 20 days after the first survey. The 7-item Likert scale used in this study ranged from strongly disagree to strongly agree.

#### Health Consciousness

Participants were then asked to complete 6 questions about their health consciousness using the Health Consciousness Scale by Jayanti and Burns [61] (Multimedia Appendix 3), which was adapted from the original Health Consciousness Scale by Kraft and Goodell [64]. The development of the health consciousness scale was facilitated by borrowing items from the literature to generate items for scales. Multiple items were used to measure each of the constructs proposed, with purification steps taken during the development of the scales. The 7-item Likert scale used in this study ranged from strongly disagree to strongly agree. Types of health consciousness questions the participants encountered include “I am interested in information about my health” and “I read more health-related articles than I did 3 years ago.”

#### Health Motivation

Participants were then asked to complete questions about their HM using the Health Motivation Scale by Jayanti and Burns [61] (Multimedia Appendix 4). Scale development was facilitated by borrowing items from the literature, and generating items for scales was used to develop the HM scale. The scale development and purification followed well-established procedures reported in the literature. This section consists of 6 questions using a 7-point Likert scale ranging from strongly disagree to strongly agree. Participants answered questions about their HM, such as, “I try to prevent common health problems before I feel any symptoms” and “I would rather enjoy life than try to make sure I am not exposing myself to health risks.”

#### Personality Traits

Finally, participants were asked to answer personality questions that generally described them as they were now and not as they wished to be in the future. The participants completed the Mini-IPIP Scale by Donnellan et al [82] (Multimedia Appendix 5), which consists of 20 questions focusing on extraversion, agreeableness, conscientiousness, neuroticism, and intellect. The stability of the Mini-IPIP Scale was measured at multiple intervals. The initial study was conducted at intervals of a few weeks, and the subsequent study was conducted over several months. The questions were answered using a 7-point Likert scale ranging from extremely inaccurate to extremely accurate. Participants rated the accuracy of statements, such as, “Am the life of the party” and “Am not really interested in others.”

#### Perceived Persuasiveness

After answering the psychological questions, participants were asked to complete questions about the perceived persuasiveness of the individual mHealth screens. The participants completed the Perceived Persuasiveness Scale by Lehto et al [94] (Multimedia Appendix 6), which consists of 3 questions using a 7-point Likert scale ranging from strongly disagree to strongly agree. During the development of the scale, data examining perceived persuasiveness were collected from participants at baseline and 2 and 6 weeks after the intervention. During the study, participants answered questions, at the screen level, about the perceived persuasiveness of the mHealth app screens, such as, “This mobile health screen has an influence on me” and “This mobile health screen makes me reconsider my overall health and wellness.”

## Results

### Exploratory Factor Analysis

EFA was conducted using SPSS to appraise the factor structure of the survey items. More specifically, principal component factoring using a Promax rotation was the extraction method for this analysis [95]. Kaiser normalization (eigenvalue>1) was used to determine the number of extracted factors. As the factor-loading cutoff varies in the literature, this research used a conventional liberal-to-conservative continuum, with all factor loadings of ≥0.4 being considered salient for this study, and cross-loadings >0.2 were considered for elimination [96,97].

The initial iteration of the EFA was conducted on 8 self-efficacy items, 6 health consciousness items, 6 HM items, 20 Big Five items, 3 perceived persuasiveness items, 3 intention items, 4 willingness to use items, and 4 marker variable questions (n=62). A total of 16 items (HM_1, HM_2, Big Five-Conscientiousness (R) Q8, Big Five-Conscientiousness (R) Q18, all 4 Big Five Agreeableness items, all 4 Big Five Openness items, and all 4 Big Five Neuroticism items were eliminated owing to cross-loading issues. In addition, 2 items—Big Five-Conscientiousness Q3 and Big Five-Conscientiousness Q13—were eliminated for having correlation coefficients below the threshold and failing to load properly on other items.

For the final stage, principal component factor analysis of the remaining 29 items resulted in 6 extracted 6 factors explaining 73.67% of the variance. The factor-loading matrix for the final solution is presented in Table 5. Hypotheses 4, 5, 7, and 8 were untestable because of the EFA results. Marker variables were removed from further statistical analyses after a lack of correlation was confirmed through EFA analysis.

**Table 5:** Final exploratory factor analysis results.

	Factor					
	1	2	3	4	5	6
SE^a^ Q1	0.736	—^b^	—	—	—	—
SE Q2	0.872	—	—	—	—	—
SE Q3	0.902	—	—	—	—	—
SE Q4	0.908	—	—	—	—	—
SE Q5	0.914	—	—	—	—	—
SE Q6	0.793	—	—	—	—	—
SE Q7	0.686	—	—	—	—	—
SE Q8	0.821	—	—	—	—	—
HC^c^ Q1	—	0.817	—	—	—	—
HC Q2	—	0.848	—	—	—	—
HC Q3	—	0.782	—	—	—	—
HC Q4	—	0.714	—	—	—	—
HC Q5	—	0.653	—	—	—	—
HC Q6	—	0.457	—	—	—	—
HM^d^ Q3	—	—	0.781	—	—	—
HM Q4	—	—	0.847	—	—	—
HM Q5	—	—	0.878	—	—	—
HM Q6	—	—	0.728	—	—	—
TF_PP^e^ Q1	—	—	—	0.973	—	—
TF_PP Q2	—	—	—	0.999	—	—
TF_PP Q3	—	—	—	0.989	—	—
MV^f^ 1	—	—	—	—	0.858	—
MV 2	—	—	—	—	0.821	—
MV 3	—	—	—	—	0.710	—
MV 4	—	—	—	—	0.830	—
E^g^ Q1	—	—	—	—	—	0.408
E Q6	—	—	—	—	—	0.624
E Q11	—	—	—	—	—	0.566
E Q16	—	—	—	—	—	0.768

^a^SE: self-efficacy.

^b^Not available.

^c^HC: health consciousness.

^d^HM: health motivation.

^e^TF_PP: perceived persuasiveness.

^f^MV: marker variable.

^g^E: extraversion.

### Statistical Results

Weighted scores were computed for self-efficacy, health consciousness, HM, extraversion, and perceived persuasiveness, using the final EFA factor loadings. Table 6 presents the Cronbach *α*, mean, SD, and intercorrelation among the variables included in this study.

Linear regression analysis was performed for weighted variables. A total of 2 linear regression models were used. Table 7 shows the regression coefficients. Model 1 included the demographic control variables of gender, age, and education level as predictors of perceived persuasiveness. The demographic variables were dummy coded with “male,” “under 40,” and “less than high school” as the reference category for gender, age, and education level. The *F* test (ie, ANOVA) for model 1 was significant (*F*_9,6540_=191.806; *P*<.001), with an adjusted *R^2^* of 0.208, indicating that the demographic variables explained 20.8% of the variance in perceived persuasiveness. Gender was a significant predictor, with women having higher perceived persuasiveness (B=0.127, SE=0.048; t_6540_=2.668; *P*=.008) and nonbinary individuals having lower perceived persuasiveness (B=−2.856, SE=0.265; t_6540_=−10.767; *P*<.001) relative to men. Age was a significant predictor, with individuals aged 40 to 59 age years (B=−0.643, SE=0.069; t_6540_=−9.377; *P*<.001) and ≥60 years (B=−2.116, SE=0.059; t_6540_=−35.752; *P*<.001) having lower perceived persuasiveness relative to individuals aged ≤40 years. Education level was a significant predictor, as individuals who held associate degrees (B=−0.411, SE=0.163; t_6540_=−2.514; *P*=.01) and Bachelor’s degrees (B=−0.581, SE=0.157; t_6540_=−3.696; *P*<.001) tended to have lower perceived persuasiveness than individuals who had not completed high school.

In model 2, the theorized effects were added as predictors. The *F* test for model 2 was significant (*F*_13,6536_=341.035; *P*<.001), with an adjusted *R^2^* of 0.403, indicating that the demographic variables self-efficacy, health consciousness, HM, and extraversion together explained 40.3% of the variance in perceived persuasiveness. Table 7 presents the regression coefficients for model 2. The nonbinary category of sex remained a significant predictor; however, the female sex category was no longer significant in model 2 (B=−0.002, SE=0.042; t_6536_=−0.048; *P*=.96). Both age categories were significant predictors in model 2. The associate and Bachelor’s categories of education level remained significant predictors in model 2, and the categories of some college (B=−0.462, SE=0.137; t_6536_=−3.378; *P*<.001) and graduate degree (B=−0.555, SE=0.139; t_6536_=−3.985; *P*<.001) became significant in model 2. Self-efficacy was a significant positive predictor (B=0.263, SE=0.026; t_6536_=10.174; *P*<.001), indicating that individuals with higher self-efficacy tended to have higher perceived persuasiveness. Health consciousness was a significant positive predictor (B=0.883, SE=0.022; t_6536_=40.000; *P*<.001), indicating that individuals with higher health consciousness tended to have higher perceived persuasiveness. HM was a significant positive predictor (B=0.200, SE=0.017; t_6536_=11.597; *P*<.001), indicating that individuals with higher HM tended to have higher perceived persuasiveness. Extraversion was a significant positive predictor (B=0.150, SE=0.026; t_6536_=5.884; *P*<.001), indicating that individuals with higher extraversion tended to have higher perceived persuasiveness. The results of significant hypothesis testing are summarized in Table 8.

**Table 6:** Correlation matrix for weighted variables^a^.

Variable	Mean (SD)	Cronbach *α*	1	2	3	4	5	6
Self-efficacy	4.574 (0.852)	.939	.833	—^b^	—	—	—	—
Health consciousness	3.455 (0.994)	.858	.239^c^	.724	—	—	—	—
Health motivation	3.071 (1.205)	.862	.067^c^	−0.132^c^	.811	—	—	—
Extraversion	2.110 (0.816)	.699	.263^c^	.142^c^	−0.069^c^	.605	—	—
Perceived persuasiveness	3.822 (2.047)	.977	.283^c^	.529^c^	.081^c^	.159^c^	.987	—
Marker variable	3.089 (1.135)	.840	.153^c^	.391^c^	.303^c^	−0.030^d^	.363^c^	.807

^a^Values on the diagonal are the square roots of the average variance extracted.

^b^Not available.

^c^*P*<.01.

^d^*P*<.05.

**Table 7:** Results for multiple linear regression modelsa (N=6550).

Variable		Model 1^a^				Model 2^b^		
		B (SE)	*t* test (*df*)	Significance (*P* value)	B (SE)		*t* test (*df*)	Significance (*P* value)
Constant		5.406 (0.161)	33.531 (6540)	<.001	0.005 (0.202)		0.023 (6537)	.98
**Control variables**								
	Gender (female)	0.127 (0.048)	2.668 (6540)	.008	−0.002 (0.042)		−0.048 (6537)	.96
	Gender (nonbinary)	−2.856 (0.265)	−10.767 (6540)	<.001	−2.239 (0.238)		−9.412 (6537)	<.001
	Age (40-59 years)	−0.643 (0.069)	−9.377 (6540)	<.001	−0.477 (0.061)		−7.869 (6537)	<.001
	Age (≥60 years)	−2.116 (0.059)	−35.752 (6540)	<.001	−1.388 (0.054)		−25.816 (6537)	<.001
	Education (high school graduate)	−0.302 (0.161)	−1.880 (6540)	.06	−0.218 (0.140)		−1.555 (6537)	.120
	Education (some college, no degree)	−0.279 (0.156)	−1.782 (6540)	.08	−0.462 (0.137)		−3.378 (6537)	<.001
	Education (associate degree)	−0.411 (0.163)	−2.514 (6540)	.01	−0.389 (0.142)		−2.731 (6537)	.006
	Education (Bachelor’s degree)	−0.581 (0.157)	−3.696 (6540)	<.001	−0.624 (0.137)		−4.542 (6537)	<.001
	Education (graduate degree)	−0.059 (0.159)	−0.370 (6540)	.71	−0.555 (0.139)		−3.985 (6537)	<.001
**Theorized effects**								
	Self-efficacy	—^c^	—	—	0.263 (0.026)		10.174 (6537)	<.001
	Health consciousness	—	—	—	0.883 (0.022)		40.000 (6537)	<.001
	Health motivation	—	—	—	0.200 (0.017)		11.597 (6537)	<.001
	Extraversion	—	—	—	0.150 (0.026)		5.884 (6537)	<.001

^a^Model 1: *R*^2^=0.208

^b^Model 2: *R*^2^=0.403.

^c^—: indicates that the theorized effects weren't added until model 2.

**Table 8:** Results of tested hypotheses.

Hypothesis	Result
Hypothesis 1: self-efficacy will positively influence interpreted mHealth screen perceived persuasiveness.	Supported
Hypothesis 2: health consciousness will positively influence interpreted mHealth screen perceived persuasiveness.	Supported
Hypothesis 3: health motivation will positively influence interpreted mHealth screen perceived persuasiveness.	Supported
Hypothesis 6: extraversion will positively influence interpreted mHealth screen perceived persuasiveness.	Supported

## Discussion

### Principal Findings

To the best of our knowledge, this study is the first to use a combination of self-efficacy, health consciousness, HM, extraversion, gender, age, and education to examine their impact on the effective engagement of users of digital health technologies. By integrating psychological characteristics, this study advances the current understanding of how psychological characteristics affect the perceived persuasiveness of persuasive technology. To evaluate this, the researchers examined the impact of psychological characteristics (self-efficacy, health consciousness, HM, and Big Five personality traits) on the perceived persuasiveness of digital health technologies. Using the PSD framework, this study was designed to evaluate how these psychological characteristics affect the perceived persuasiveness of digital health technologies. In addition, the dynamic intertwining of psychological characteristics that drives the perceived persuasiveness of the primary PSD technique categories was illuminated through multiple linear regression analysis.

Furthermore, this study opens a pathway for designers of digital health technologies to gain further knowledge on why individual characteristics must be considered during the design process. Keizer et al [42] suggested that misalignment between end users and digital technologies is often a result of developers failing to consider the end user during the development process. Although the benefits of personalizing persuasive systems have been acknowledged, the field is still in its infancy, and there is very little knowledge on the best way to tailor these technologies [98,99]. The findings from this study suggest that using a dynamic, data-centered approach that considers that the end users’ self-efficacy, health consciousness, HM, extraversion, age, gender, and education could be a way to increase the perceived persuasiveness of digital health technologies.

In addition, this research offers developers vital information pertaining to user-centric development of persuasive digital health technologies. The information gained can be used by designers to increase the perceived persuasiveness of digital health technologies by providing guidance on how to dynamically use PSD principles based on an individual’s psychological characteristics and demographic makeup. These PSD principles can be delivered in various components such as virtual reality or health care gaming approaches, which can further establish a stronger connection to an individual’s psychological characteristics [100].

On the basis of these major findings, the role of self-efficacy should be considered by persuasive technology designers. Statistical analysis found self-efficacy to be a significant positive predictor of perceived persuasiveness. Multiple linear regression analyses found that health consciousness was a significant positive predictor of perceived persuasiveness. In addition, the model found HM to be a significant positive predictor of perceived persuasiveness. Multiple linear regression analyses also found extraversion to be a significantly positive predictor of perceived persuasiveness. These findings are important, as they shed additional light on which psychological characteristics influence a user’s perceived persuasiveness. In addition, it helps validate why one-size-fits-all approaches do not necessarily work. The findings suggest that individuals with low self-efficacy and low health consciousness will not necessarily be influenced (perceived persuasiveness) by the same mHealth app design as those with higher self-efficacy and health consciousness levels.

Demographic data, such as age and gender, should also be considered by developers of digital health technologies. The findings strongly suggest that the distribution of perceived persuasiveness shifts from negatively skewed to positively skewed as an individual ages. In addition, this shift occurs earlier in women (ie, aged 40-59 years) than in men who do not shift until the oldest age group (ie, aged ≥60 years). The perceived persuasiveness by age group and gender is available in Multimedia Appendix 7. This was an interesting and unexpected finding, and additional research is required. Potentially, these findings can represent the aging process for which health consciousness, for example, has increased owing to typical chronic diseases that manifest as individuals age.

### Future Research and Limitations

Despite the theoretical and practical contributions of this study, there are limitations to the generalizability of the findings. Further examination of the demographic data showed that only 7.3% (19/262) of participants were between the ages of 18 and 29 years. Additional research should be conducted that focuses on the younger population, aged 18 to 29 years.

The research only examined extraversion due to multicollinearity issues with other items from the Big Five personality traits. Sleep et al [101] found that longer measures contain considerably more variance than shorter, more condensed measures. Further studies should use a more extensive Big Five personality test such as the Neo Personality Inventory [102] rather than the Mini-IPIP Scale [82].

The Adult Hope Scale by Snyder et al [103] was also dropped from the study owing to multicollinearity issues with the New General Self-Efficacy Scale by Chen et al [93]. It was observed that all the self-efficacy constructs and adult hope constructs were cross-loading; therefore, adult hope was eliminated because self-efficacy is regarded as a core premise of human performance across multiple domains, and adult hope measurements conceptually and operationally function synonymously as self-efficacy [104].

Multicollinearity issues were also identified among perceived persuasiveness, intention, and willingness to use; therefore, the intention and willingness to use constructs were eliminated from the model because perceived persuasiveness was studied across multiple domains, and perceived persuasiveness was more pursuant to this study.

A key limitation of this study is the use of static screens. A fully developed app will allow researchers to evaluate the engagement of digital health tools. Running these studies in tandem will allow researchers to evaluate engagement on both sides to see if higher perceived persuasiveness leads to higher engagement.

### Conclusions

This study aimed to examine how users’ psychological characteristics influence the perceived persuasiveness of digital health technologies. This research contributes to advancing the field of data-driven, user-centric development of persuasive technologies by investigating the intertwining of users’ psychological characteristics and the perceived persuasiveness of digital health technologies. This work opens a new research avenue by examining the role of psychological characteristics in interpreting the perceived persuasiveness of mHealth screens. The use of dynamic data-driven capabilities is important for advancing perceived persuasiveness, which has the potential to engage users of digital health technologies successfully.

This work also describes the roles that psychological characteristics play in interpreting mHealth screen perceived persuasiveness. Evidence has shown that self-efficacy, health consciousness, HM, extraversion, gender, age, and education significantly influence the perceived persuasiveness of digital health technologies. Moreover, this study showed that varying combinations of psychological characteristics and demographic variables affected the perceived persuasiveness of the primary persuasive technology category. Incorporating these psychological characteristics and demographic variables should allow digital health technology developers to overcome the gap stemming from one-size-fits-all approaches.

On the basis of the findings of this research, mHealth app researchers and developers should design apps that dynamically interact with users using psychological characteristics and demographics to drive the persuasive techniques presented to the user. This process should include a pre-enrollment assessment, for which the user’s psychological characteristics are evaluated before deployment of the mHealth app. This would allow for the right persuasive techniques to be deployed in an attempt to better engage the user, which can potentially lead to more favorable behavior. Moving from a “one-size-fits-all” to a personalized persuasive approach has the potential to create long-term engagement, which has plagued mHealth researchers and developers.

## Appendix

Multimedia Appendix 1 Developed mobile health screens.

Multimedia Appendix 2 New General Self-Efficacy Scale.

Multimedia Appendix 3 Health Consciousness Scale.

Multimedia Appendix 4 Health Motivation Scale.

Multimedia Appendix 5 Big Five Mini-International Personality Item Pool Scale.

Multimedia Appendix 6 Perceived Persuasiveness Scale.

Multimedia Appendix 7 Perceived persuasiveness by age and gender.

## Abbreviations

EFA: exploratory factor analysis
HM: health motivation
mHealth: mobile health
Mini-IPIP: Mini-International Personality Item Pool
PSD: persuasive system design

## References

[1] Matthews J, Win KT, Oinas-Kukkonen H, Freeman M (2016). Persuasive technology in mobile applications promoting physical activity: a systematic review. J Med Syst.

[2] Birnbaum F, Lewis D, Rosen RK, Ranney ML (2015). Patient engagement and the design of digital health. Acad Emerg Med.

[3] O'Brien H, Filimowicz M, Tzankova V (2018). A holistic approach to measuring user engagement. New Directions in Third Wave Human-Computer Interaction: Volume 2 - Methodologies.

[4] Taki S, Lymer S, Russell CG, Campbell K, Laws R, Ong KL, Elliott R, Denney-Wilson E (2017). Assessing user engagement of an mHealth intervention: development and implementation of the growing healthy app engagement index. JMIR Mhealth Uhealth.

[5] O'Brien HL, Toms EG (2008). What is user engagement? A conceptual framework for defining user engagement with technology. J Am Soc Inf Sci.

[6] Vandelanotte C, Müller AM, Short CE, Hingle M, Nathan N, Williams SL, Lopez ML, Parekh S, Maher CA (2016). Past, present, and future of eHealth and mHealth research to improve physical activity and dietary behaviors. J Nutr Educ Behav.

[7] Orji R, Moffatt K (2018). Persuasive technology for health and wellness: state-of-the-art and emerging trends. Health Informatics J.

[8] Silva M, Graham F, Levack W, Hay-Smith J (2019). Persuasive technology and behaviour change in parent-focused eHealth interventions supporting child health: a scoping review protocol. N Z J Physiother.

[9] Wall HJ, Campbell CC, Kaye LK, Levy A, Bhullar N (2019). Personality profiles and persuasion: an exploratory study investigating the role of the Big-5, type D personality and the Dark Triad on susceptibility to persuasion. Pers Individ Dif.

[10] Kaptein M, Markopoulos P, de Ruyter B, Aarts E (2015). Personalizing persuasive technologies: explicit and implicit personalization using persuasion profiles. Int J Human Comput Stud.

[11] Engl E, Smittenaar P, Sgaier SK (2019). Identifying population segments for effective intervention design and targeting using unsupervised machine learning: an end-to-end guide. Gates Open Res.

[12] Abdullahi AM, Oyibo K, Orji R, Kawu AA (2019). The influence of age, gender, and cognitive ability on the susceptibility to persuasive strategies. Information.

[13] O'Brien HL, Arguello J, Capra R (2020). An empirical study of interest, task complexity, and search behaviour on user engagement. Inf Process Manag.

[14] Goldkuhl G (2013). From ensemble view to ensemble artefact – an inquiry on conceptualisations of the IT artefact. Syst Sign Action.

[15] van Gemert-Pijnen JE, Nijland N, van Limburg M, Ossebaard HC, Kelders SM, Eysenbach G, Seydel ER (2011). A holistic framework to improve the uptake and impact of eHealth technologies. J Med Internet Res.

[16] Michie S, Yardley L, West R, Patrick K, Greaves F (2017). Developing and evaluating digital interventions to promote behavior change in health and health care: recommendations resulting from an international workshop. J Med Internet Res.

[17] Karekla M, Kasinopoulos O, Neto DD, Ebert DD, Van Daele T, Nordgreen T, Höfer S, Oeverland S, Jensen KL (2019). Best practices and recommendations for digital interventions to improve engagement and adherence in chronic illness sufferers. Eur Psychol.

[18] Orji R, Oyibo K, Lomotey RK, Orji FA (2019). Socially-driven persuasive health intervention design: competition, social comparison, and cooperation. Health Informatics J.

[19] Iyengar MS, Florez-Arango JF, Garcia CA (2009). GuideView: a system for developing structured, multimodal, multi-platform persuasive applications. Proceedings of the 4th International Conference on Persuasive Technology.

[20] Thomson C, Nash J, Maeder A (2016). Persuasive design for behaviour change apps: issues for designers. Proceedings of the Annual Conference of the South African Institute of Computer Scientists and Information Technologists.

[21] Tuman M, Moyer A (2019). Health intentions and behaviors of health app owners: a cross-sectional study. Psychol Health Med.

[22] Al-Ramahi M, El-Gayar O, Liu J (2016). Discovering design principles for persuasive systems: a grounded theory and text mining approach. Proceedings of the 49th Hawaii International Conference on System Sciences.

[23] Alkhaldi G, Hamilton FL, Lau R, Webster R, Michie S, Murray E (2016). The effectiveness of prompts to promote engagement with digital interventions: a systematic review. J Med Internet Res.

[24] Holdener M, Gut A, Angerer A (2020). Applicability of the user engagement scale to mobile health: a survey-based quantitative study. JMIR Mhealth Uhealth.

[25] Zagalo N, Zagalo N (2020). From experience to engagement. Engagement Design: Designing for Interaction Motivations.

[26] Sahin C (2018). Rules of engagement in mobile health: what does mobile health bring to research and theory?. Contemp Nurse.

[27] Helsper EJ, Eynon R (2013). Distinct skill pathways to digital engagement. Eur J Commun.

[28] Salehzadeh Niksirat K, Sarcar S, Sun H, Law EL, Clemmensen T, Bardzell J, Oulasvirta A, Silpasuwanchai C, Light A, Ren X (2018). Approaching engagement towards human-engaged computing. Extended Abstracts of the 2018 CHI Conference on Human Factors in Computing Systems.

[29] Chapman P, Selvarajah S, Webster J (1999). Engagement in multimedia training systems. Proceedings of the 32nd Annual Hawaii International Conference on Systems Sciences.

[30] Tarute A, Nikou S, Gatautis R (2017). Mobile application driven consumer engagement. Telemat Inform.

[31] Wiafe I, Khosrow-Pour M (2018). The role of U-FADE in selecting persuasive system features. Encyclopedia of Information Science and Technology. 4th edition.

[32] Gena C, Grillo P, Lieto A, Mattutino C, Vernero F (2019). When personalization is not an option: an in-the-wild study on persuasive news recommendation. Information.

[33] Orji R, Mandryk RL, Vassileva J (2015). Gender, age, and responsiveness to Cialdini’s persuasion strategies. Proceedings of the 10th International Conference on Persuasive Technology.

[34] Orji R, Vassileva J, Mandryk RL (2014). Modeling the efficacy of persuasive strategies for different gamer types in serious games for health. User Model User Adap Interact.

[35] Berkovsky S, Freyne J, Oinas-Kukkonen H (2012). Influencing individually: fusing personalization and persuasion. ACM Trans Interact Intell Syst.

[36] Orji FA, Oyibo K, Orji R, Greer J, Vassileva J (2019). Personalization of persuasive technology in higher education. Proceedings of the 27th ACM Conference on User Modeling, Adaptation and Personalization.

[37] Ruijten PA (2021). The similarity-attraction paradigm in persuasive technology: effects of system and user personality on evaluations and persuasiveness of an interactive system. Behav Inf Technol.

[38] Orji RO, Vassileva J, Mandryk RL (2013). Modeling gender differences in healthy eating determinants for persuasive intervention design. Proceedings of the 8th International Conference on Persuasive Technology.

[39] Taype GE, Calani MC (2020). Extending persuasive system design frameworks: an exploratory study. Proceedings of Information Technology and Systems.

[40] Almunawar MN, Anshari M, Younis MZ (2015). Incorporating customer empowerment in mobile health. Health Policy Technol.

[41] Yardley L, Choudhury T, Patrick K, Michie S (2016). Current issues and future directions for research into digital behavior change interventions. Am J Prev Med.

[42] Keizer J, Beerlage-de Jong N, al Naiemi N, van Gemert-Pijnen LJ (2020). Persuading from the start: participatory development of sustainable persuasive data-driven technologies in healthcare. Proceedings of the 15th International Conference on Persuasive Technology.

[43] Riley WT, Rivera DE, Atienza AA, Nilsen W, Allison SM, Mermelstein R (2011). Health behavior models in the age of mobile interventions: are our theories up to the task?. Transl Behav Med.

[44] Nahum-Shani I, Smith SN, Spring BJ, Collins LM, Witkiewitz K, Tewari A, Murphy SA (2018). Just-in-Time Adaptive Interventions (JITAIs) in mobile health: key components and design principles for ongoing health behavior support. Ann Behav Med.

[45] Shin Y, Kim J (2018). Data-centered persuasion: nudging user's prosocial behavior and designing social innovation. Comput Human Behav.

[46] Dalecke S, Karlsen R (2020). Designing dynamic and personalized nudges. Proceedings of the 10th International Conference on Web Intelligence, Mining and Semantics.

[47] Abdullahi AM, Orji R, Kawu AA (2019). Gender, age and subjective well-being: towards personalized persuasive health interventions. Information.

[48] Bandura A (1997). Self-Efficacy: The Exercise of Control.

[49] Bong M (1997). Generality of academic self-efficacy judgments: evidence of hierarchical relations. J Educ Psychol.

[50] van Dinther M, Dochy F, Segers M, Braeken J (2013). The construct validity and predictive validity of a self-efficacy measure for student teachers in competence-based education. Stud Educ Eval.

[51] Bulfone G, Badolamenti S, Biagioli V, Maurici M, Macale L, Sili A, Vellone E, Alvaro R (2021). Nursing students' academic self-efficacy: a longitudinal analysis of academic self-efficacy changes and predictive variables over time. J Adv Nurs.

[52] Simonavice EM, Wiggins MS (2008). Exercise barriers, self-efficacy, and stages of change. Percept Mot Skills.

[53] Koring M, Richert J, Lippke S, Parschau L, Reuter T, Schwarzer R (2012). Synergistic effects of planning and self-efficacy on physical activity. Health Educ Behav.

[54] Falco LD, Summers JJ (2017). Improving career decision self-efficacy and STEM self-efficacy in high school girls: evaluation of an intervention. J Career Dev.

[55] Messina R, Rucci P, Sturt J, Mancini T, Fantini MP (2018). Assessing self-efficacy in type 2 diabetes management: validation of the Italian version of the Diabetes Management Self-Efficacy Scale (IT-DMSES). Health Qual Life Outcomes.

[56] Bandura A (1977). Self-efficacy: toward a unifying theory of behavioral change. Psychol Rev.

[57] Medrano LA, Flores-Kanter E, Moretti L, Pereno GL (2016). Effects of induction of positive and negative emotional states on academic self-efficacy beliefs in college students. Psicología Educativa.

[58] Buckworth J (2017). Promoting self-efficacy for healthy behaviors. ACSM'S Health Fitness J.

[59] Lindenmeier J (2008). Promoting volunteerism: effects of self-efficacy, advertisement-induced emotional arousal, perceived costs of volunteering, and message framing. Voluntas.

[60] Wigal JK, Creer TL, Kotses H (1991). The COPD self-efficacy scale. Chest.

[61] Jayanti RK, Burns AC (1998). The antecedents of preventive health care behavior: an empirical study. J Acad Market Sci.

[62] Yan M, Filieri R, Raguseo E, Gorton M (2021). Mobile apps for healthy living: factors influencing continuance intention for health apps. Technol Forecast Soc Change.

[63] Chen MF, Lin NP (2018). Incorporation of health consciousness into the technology readiness and acceptance model to predict app download and usage intentions. Internet Res.

[64] Kraft FB, Goodell PW (1993). Identifying the health conscious consumer. J Health Care Mark.

[65] Barauskaite D, Gineikiene J, Fennis BM, Auruskeviciene V, Yamaguchi M, Kondo N (2018). Eating healthy to impress: how conspicuous consumption, perceived self-control motivation, and descriptive normative influence determine functional food choices. Appetite.

[66] Parashar S, Mungra Y, Sood G (2019). Health consciousness as an enabler for exploratory buying behavior among consumers. SCMS J Indian Manag.

[67] Ahadzadeh AS, Pahlevan Sharif S, Sim Ong F (2018). Online health information seeking among women: the moderating role of health consciousness. Online Inf Rev.

[68] Donalds C, Osei-Bryson KM (2020). Cybersecurity compliance behavior: exploring the influences of individual decision style and other antecedents. Int J Inf Manag.

[69] Park J, Ahn J, Yoo WS (2017). The effects of price and health consciousness and satisfaction on the medical tourism experience. J Healthc Manag.

[70] Tanner EC, Vann RJ, Kizilova E (2020). Consumer-level perceived access to health services and its effects on vulnerability and health outcomes. J Public Policy Market.

[71] Toste JR, Didion L, Peng P, Filderman MJ, McClelland AM (2020). A meta-analytic review of the relations between motivation and reading achievement for K–12 students. Rev Educ Res.

[72] Dehghani M, Kim KJ, Dangelico RM (2018). Will smartwatches last? Factors contributing to intention to keep using smart wearable technology. Telemat Inform.

[73] Ferron JC, Elbogen EB, Swanson JW, Swartz MS, McHugo GJ (2010). A conceptually based scale to measure consumers’ treatment motivation. Res Soc Work Pract.

[74] Ryan RM, Deci EL (2020). Intrinsic and extrinsic motivation from a self-determination theory perspective: definitions, theory, practices, and future directions. Contemp Educ Psychol.

[75] Muenks K, Wigfield A, Eccles JS (2018). I can do this! The development and calibration of children’s expectations for success and competence beliefs. Dev Rev.

[76] Wagner 3rd B, Liu E, Shaw SD, Iakovlev G, Zhou L, Harrington C, Abowd G, Yoon C, Kumar S, Murphy S, Spring B, Nahum-Shani I (2017). e wrapper: operationalizing engagement strategies in mHealth. Proc ACM Int Conf Ubiquitous Comput.

[77] Roccas S, Sagiv L, Schwartz SH, Knafo A (2002). The Big Five personality factors and personal values. Pers Soc Psychol Bull.

[78] Goldberg LR (1990). An alternative "description of personality": the big-five factor structure. J Pers Soc Psychol.

[79] Costa Jr PT, McCrae RR (1992). Revised NEO Personality Inventory (NEO-PI-R) and NEO Five-Factor Inventory (NEO-FFI) Professional Manual.

[80] Borghans L, Duckworth AL, Heckman JJ, ter Weel B (2008). The economics and psychology of personality traits. J Human Resources.

[81] Gosling SD, Rentfrow PJ, Swann Jr WB (2003). A very brief measure of the Big-Five personality domains. J Res Pers.

[82] Donnellan MB, Oswald FL, Baird BM, Lucas RE (2006). The mini-IPIP scales: tiny-yet-effective measures of the Big Five factors of personality. Psychol Assess.

[83] Wilmot MP, Wanberg CR, Kammeyer-Mueller JD, Ones DS (2019). Extraversion advantages at work: a quantitative review and synthesis of the meta-analytic evidence. J Appl Psychol.

[84] White JK, Hendrick SS, Hendrick C (2004). Big five personality variables and relationship constructs. Pers Individ Dif.

[85] Stajkovic AD, Bandura A, Locke EA, Lee D, Sergent K (2018). Test of three conceptual models of influence of the big five personality traits and self-efficacy on academic performance: a meta-analytic path-analysis. Pers Individ Dif.

[86] Nolan A, McCrory C, Moore P (2019). Personality and preventive healthcare utilisation: evidence from the Irish Longitudinal Study on Ageing. Prev Med.

[87] (2019). Qualtrics.

[88] Pangbourne K, Bennett S, Baker A (2020). Persuasion profiles to promote pedestrianism: effective targeting of active travel messages. Travel Behav Soc.

[89] Kniffin KM, Bogan VL, Just DR (2019). "Big men" in the office: the gender-specific influence of weight upon persuasiveness. PLoS One.

[90] Oinas-Kukkonen H, Harjumaa M (2009). Persuasive systems design: key issues, process model, and system features. Commun Assoc Inf Syst.

[91] Rosenzweig E (2015). Successful User Experience: Strategies and Roadmaps.

[92] (2019). Buildfire Corporation.

[93] Chen G, Gully SM, Eden D (2001). Validation of a new general self-efficacy scale. Organ Res Method.

[94] Lehto T, Oinas-Kukkonen H, Drozd F (2012). Factors affecting perceived persuasiveness of a behavior change support system. Proceedings of the 33rd International Conference on Information Systems.

[95] Williams B, Onsman A, Brown T (2010). Exploratory factor analysis: a five-step guide for novices. Australas J Paramed.

[96] Knekta E, Runyon C, Eddy S (2019). One size doesn't fit all: using factor analysis to gather validity evidence when using surveys in your research. CBE Life Sci Educ.

[97] Matsunaga M (2010). How to factor-analyze your data right: do’s, don’ts, and how-to’s. Int J Psychol Res.

[98] Orji R, Kaptein M, Ham J, Oyibo K, Nwokeji J (2018). Personalizing persuasive technologies: a road map to the future. Proceedings of the 13th International Conference on Persuasive Technology.

[99] Oyibo K, Orji R, Ham J, Nwokeji J, Ciocarlan A (2019). Personalizing persuasive technologies: personalization for wellbeing. Personalizing Persuasive Technology Workshop.

[100] Horsham C, Dutton-Regester K, Antrobus J, Goldston A, Price H, Ford H, Hacker E (2021). A virtual reality game to change sun protection behavior and prevent cancer: user-centered design approach. JMIR Serious Games.

[101] Sleep CE, Lynam DR, Miller JD (2021). A comparison of the validity of very brief measures of the Big Five/Five-Factor Model of personality. Assessment.

[102] McCrae RR, Costa Jr PT, Martin TA (2005). The NEO-PI-3: a more readable revised NEO Personality Inventory. J Pers Assess.

[103] Snyder CR, Harris C, Anderson JR, Holleran SA, Irving LM, Sigmon ST (1991). Adult Hope Scale (AHS). J Pers Soc Psychol.

[104] Zhou M, Kam CC (2016). Hope and general self-efficacy: two measures of the same construct?. J Psychol.

