# Supplementary material for: The Intersection of Persuasive System Design and Personalization in Mobile Health: Statistical Evaluation
Source: JMIR Mhealth Uhealth. 2022 Sep 14;10(9):e40576. doi: 10.2196/40576 (PMC9520383; doi:10.2196/40576)
Supplement: Multimedia Appendix 1 [file mhealth_v10i9e40576_app1.pdf]

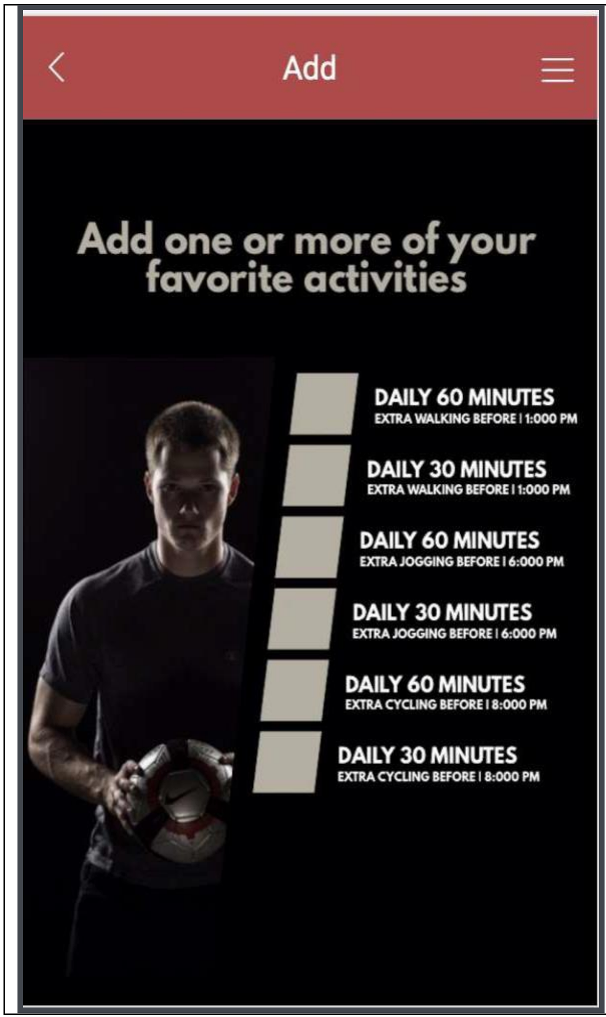
Add
Add one or more of your favorite activities
DAILY 60 MINUTES
EXTRA WALKING BEFORE I 1:000 PM
DAILY 30 MINUTES
EXTRA WALKING BEFORE I 1:000 PM
DAILY 60 MINUTES
EXTRA JOGGING BEFORE I 6:000 PM
DAILY 30 MINUTES
EXTRA JOGGING BEFORE I 6:000 PM
DAILY 60 MINUTES
EXTRA CYCLING BEFORE I 8:000 PM
DAILY 30 MINUTES
EXTRA CYCLING BEFORE I 8:000 PM

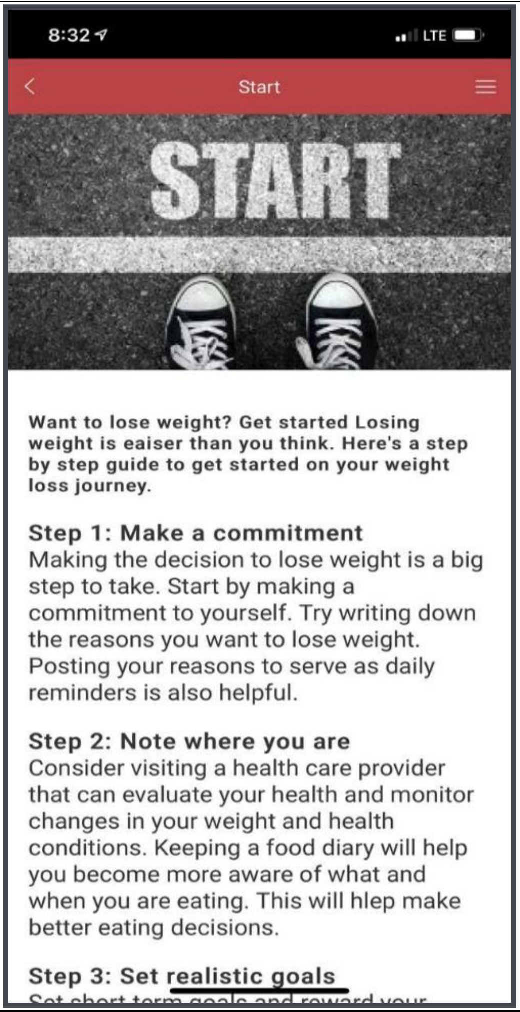
8:32
LTE
Start
START
Want to lose weight? Get started Losing weight is eaiser than you think. Here's a step by step guide to get started on your weight loss journey.
Step 1: Make a commitment
Making the decision to lose weight is a big step to take. Start by making a commitment to yourself. Try writing down the reasons you want to lose weight. Posting your reasons to serve as daily reminders is also helpful.
Step 2: Note where you are
Consider visiting a health care provider that can evaluate your health and monitor changes in your weight and health conditions. Keeping a food diary will help you become more aware of what and when you are eating. This will hlep make better eating decisions.
Step 3: Set realistic goals

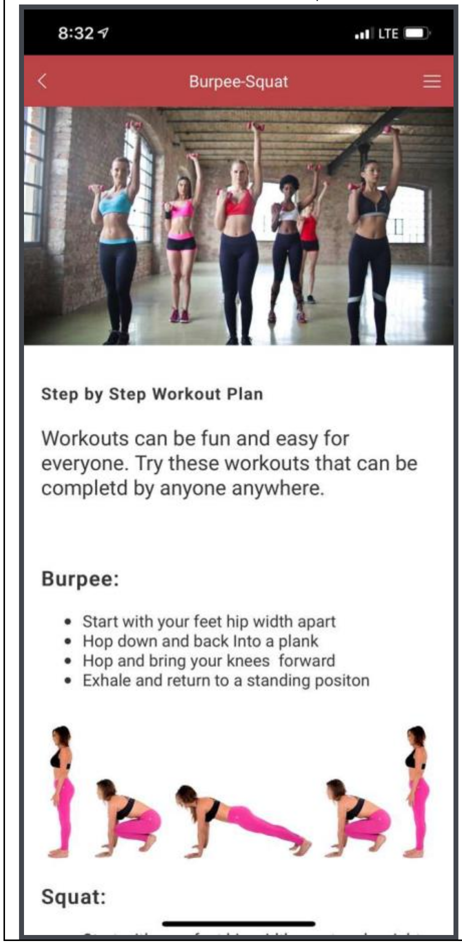	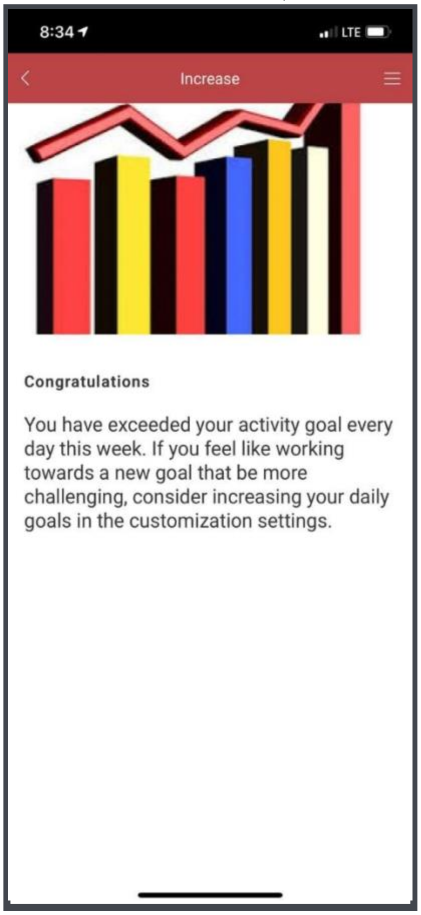

8:32
LTE
Burpee-Squat
Step by Step Workout Plan
Workouts can be fun and easy for everyone. Try these workouts that can be completd by anyone anywhere.
Burpee:
• Start with your feet hip width apart
• Hop down and back Into a plank
• Hop and bring your knees forward
• Exhale and return to a standing positon
Squat:

8:34
LTE
Increase
Congratulations
You have exceeded your activity goal every day this week. If you feel like working towards a new goal that be more challenging, consider increasing your daily goals in the customization settings.

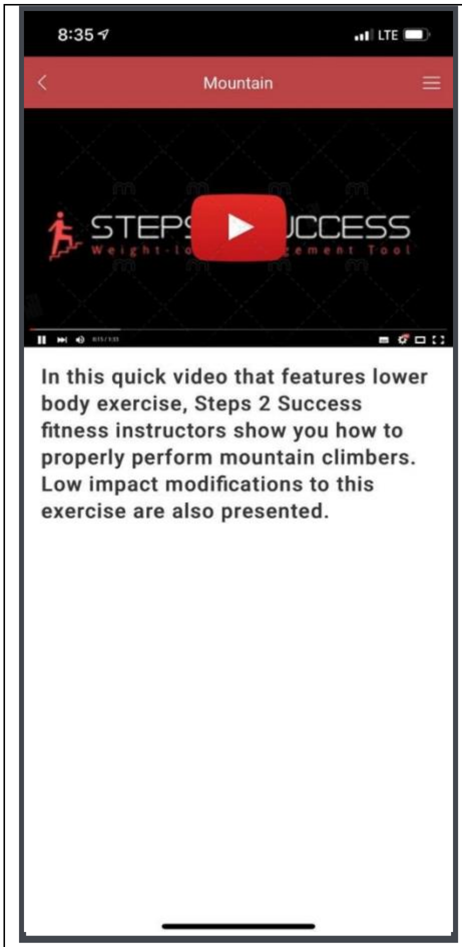	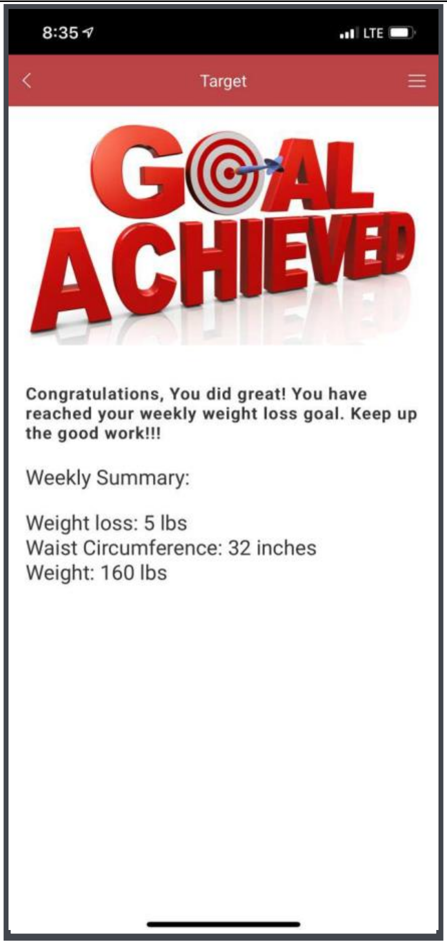

8:35
LTE
Mountain
STEPS
UCCESS
In this quick video that features lower body exercise, Steps 2 Success fitness instructors show you how to properly perform mountain climbers. Low impact modifications to this exercise are also presented.

8:35
LTE
Target
GOAL
ACHIEVED
Congratulations, You did great! You have reached your weekly weight loss goal. Keep up the good work!!!
Weekly Summary:
Weight loss: 5 lbs
Waist Circumference: 32 inches
Weight: 160 lbs

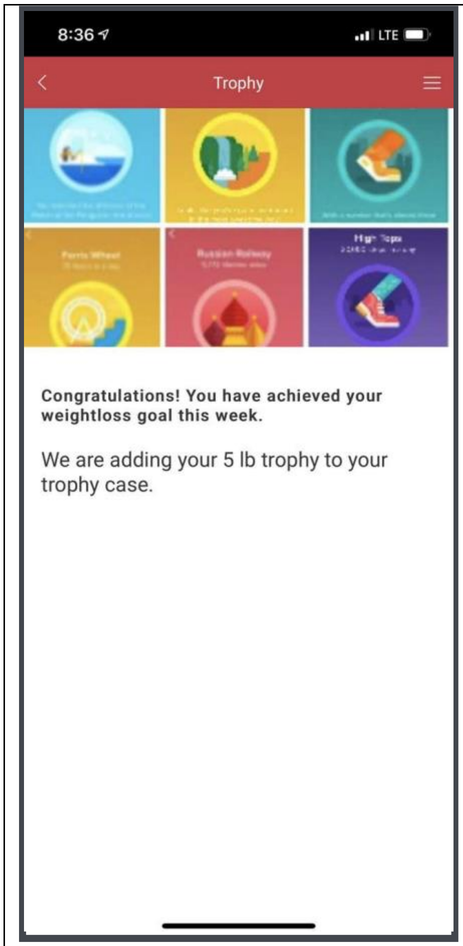
8:36
LTE
Trophy
Ferris Wheel
Russian Railway
High Tops
Congratulations! You have achieved your weightloss goal this week.
We are adding your 5 lb trophy to your trophy case.

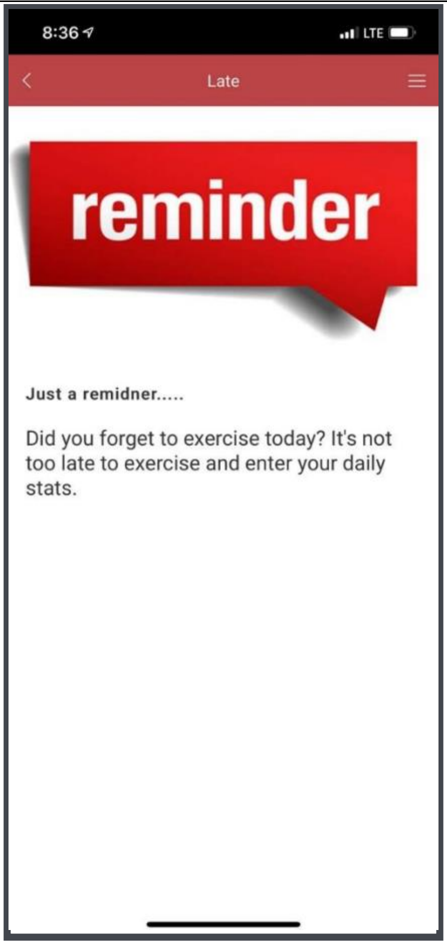
8:36
LTE
Late
reminder
Just a remidner.....
Did you forget to exercise today? It's not too late to exercise and enter your daily stats.

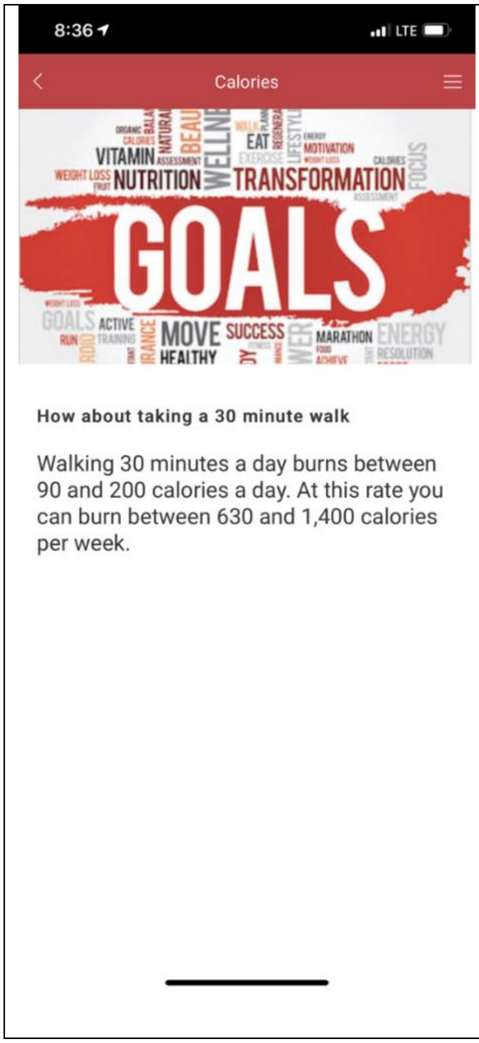
8:36
LTE
Calories
GOALS
NUTRITION
TRANSFORMATION
VITAMIN
MOVE
SUCCESS
MARATHON
ENERGY
ACTIVE
How about taking a 30 minute walk
Walking 30 minutes a day burns between 90 and 200 calories a day. At this rate you can burn between 630 and 1,400 calories per week.

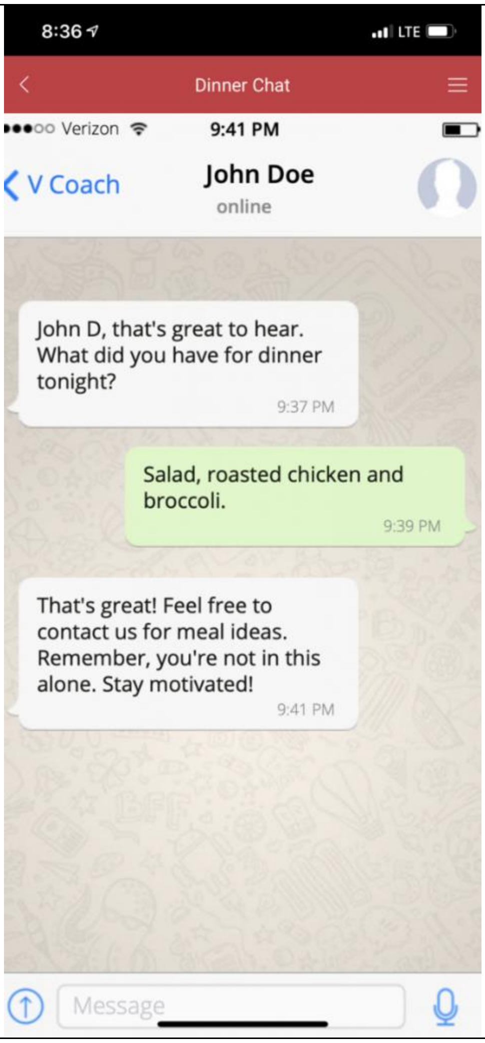
8:36
LTE
Dinner Chat
Verizon
9:41 PM
V Coach
John Doe
online
John D, that's great to hear. What did you have for dinner tonight?
9:37 PM
Salad, roasted chicken and broccoli.
9:39 PM
That's great! Feel free to contact us for meal ideas. Remember, you're not in this alone. Stay motivated!
9:41 PM
Message

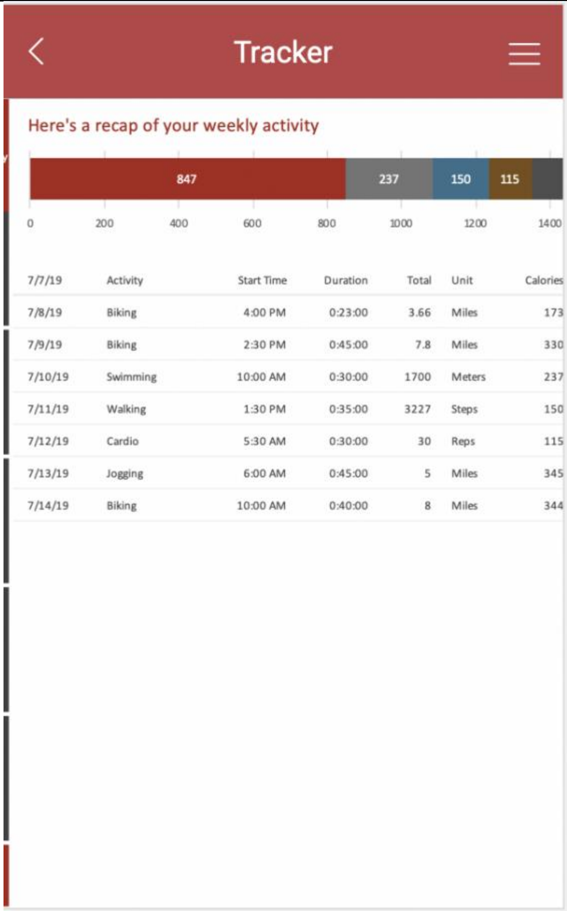

7/7/19	Activity	Start Time	Duration	Total	Unit	Calories
7/8/19	Biking	4:00 PM	0:23:00	3.66	Miles	173
7/9/19	Biking	2:30 PM	0:45:00	7.8	Miles	330
7/10/19	Swimming	10:00 AM	0:30:00	1700	Meters	237
7/11/19	Walking	1:30 PM	0:35:00	3227	Steps	150
7/12/19	Cardio	5:30 AM	0:30:00	30	Reps	115
7/13/19	Jogging	6:00 AM	0:45:00	5	Miles	345
7/14/19	Biking	10:00 AM	0:40:00	8	Miles	344

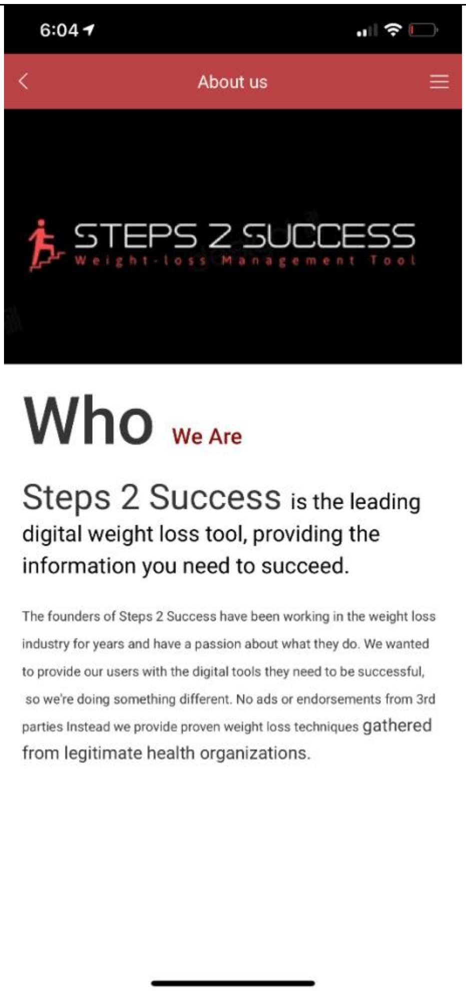

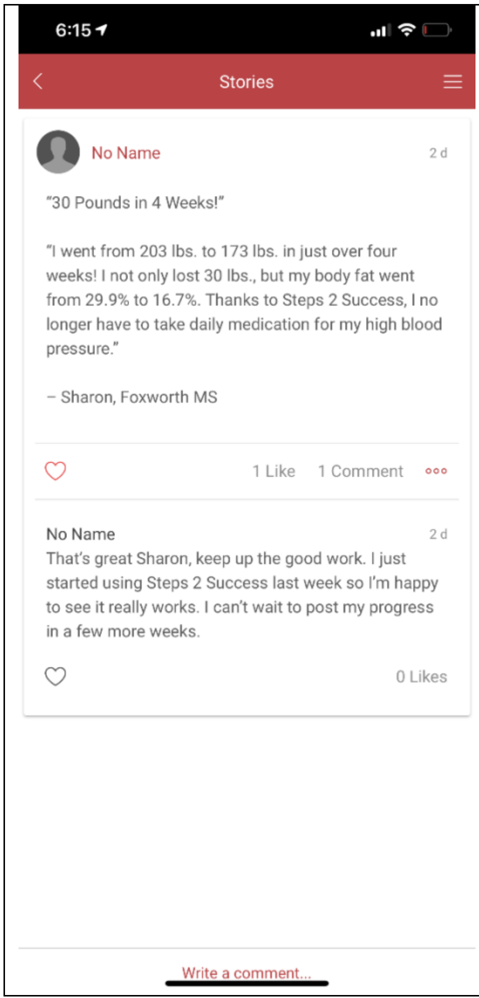
6:15
Stories
No Name
2 d
"30 Pounds in 4 Weeks!"
"I went from 203 lbs. to 173 lbs. in just over four weeks! I not only lost 30 lbs., but my body fat went from 29.9% to 16.7%. Thanks to Steps 2 Success, I no longer have to take daily medication for my high blood pressure."
– Sharon, Foxworth MS
1 Like
1 Comment
No Name
2 d
That's great Sharon, keep up the good work. I just started using Steps 2 Success last week so I'm happy to see it really works. I can't wait to post my progress in a few more weeks.
0 Likes
Write a comment...

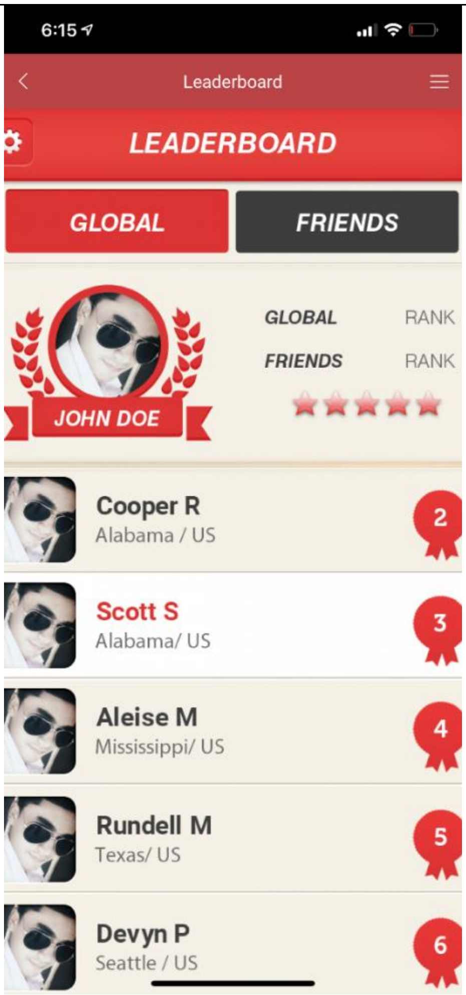
6:15
Leaderboard
LEADERBOARD
GLOBAL
FRIENDS
JOHN DOE
GLOBAL
RANK
FRIENDS
RANK
Cooper R
Alabama / US
2
Scott S
Alabama/ US
3
Aleise M
Mississippi/ US
4
Rundell M
Texas/ US
5
Devyn P
Seattle / US
6

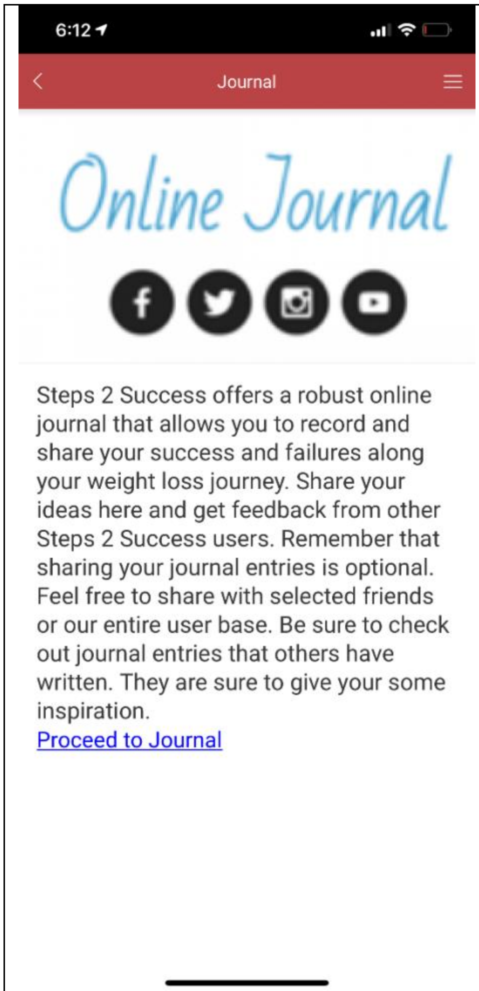
6:12
Journal
Online Journal
Steps 2 Success offers a robust online journal that allows you to record and share your success and failures along your weight loss journey. Share your ideas here and get feedback from other Steps 2 Success users. Remember that sharing your journal entries is optional. Feel free to share with selected friends or our entire user base. Be sure to check out journal entries that others have written. They are sure to give your some inspiration.
Proceed to Journal

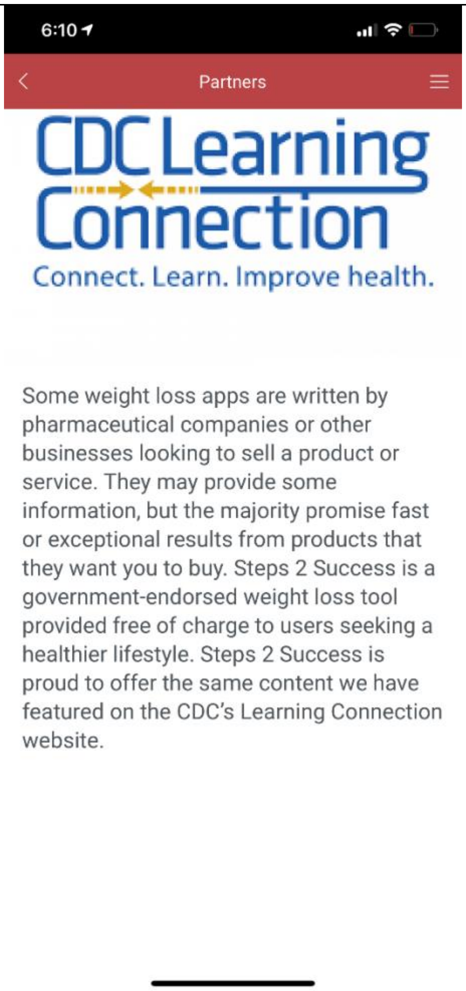
6:10
Partners
CDC Learning Connection
Connect. Learn. Improve health.
Some weight loss apps are written by pharmaceutical companies or other businesses looking to sell a product or service. They may provide some information, but the majority promise fast or exceptional results from products that they want you to buy. Steps 2 Success is a government-endorsed weight loss tool provided free of charge to users seeking a healthier lifestyle. Steps 2 Success is proud to offer the same content we have featured on the CDC's Learning Connection website.

12:34

❌ Ads

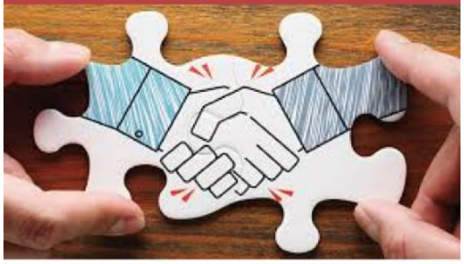

❌ Ad Free Experience

Steps 2 Success is a grant funded mHealth app that provides free assistance to users seeking to live a healthier lifestyle. We hope that Step 2 Success users find our Ad free platform useful.

1:07

Strategy

STEPS 2 SUCCESS
Weight-loss Management Tool

Steps 2 Success has implemented strategies from some of the world's leading weight loss authorities to bring you a better weight loss experience. According to the CDC, healthy weight loss should be gradual and steady. Steps 2 Success follows **these** guidelines and helps users lose weight in increments that are shown to promote long term weight loss. We want our users to be successful at keeping weight off.

2:30

Apple

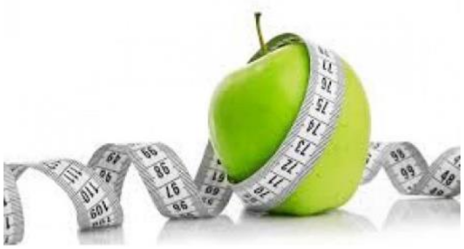

✅ **Steps 2 Success is a mHealth app that was developed to help users monitor their health and assist them in maintaining a healthy lifestyle. Steps 2 Success uses guidelines established by trusted sources to ensure our users lose weight in a safe manner. Visit https://www.cdc.gov/healthyweight for additional information about the methods used by Steps 2 Success.**

11:17 LTE

HIPAA

HIPAA COMPLIANT

HIPAA Compliance Statement

Steps 2 Success has implemented many safeguards to ensure its devices, services, websites and data systems (collectively "Products") are compliant with the regulations and conditions set forth in the Health Insurance Portability and Availability Act of 1996 (**HIPAA**):

- Electronic Transactions and Code Set Standards
- Privacy Rule
- Security Rule
- Health Information Technology for Economic and Clinical Health (HITECH) Act
- Omnibus Final Rule

For more information regarding our privacy practices, read our Notice of Privacy Practices.

11:18 LTE

Contact

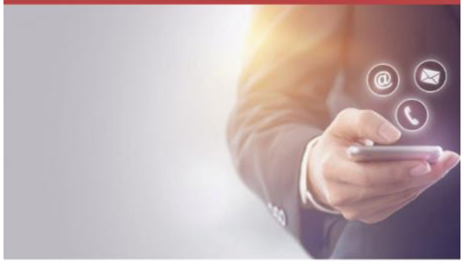

Contact Us - Steps 2 Success

Get answers to your questions and share your knowledge with other custormers.

Chat - We are online 24/7
Chat With Us

Call Now
800-800-0000

Tweet Us
Our favorite way to interact with our fans and customers all day, every day. Tweet us
@steps2success

Visit the Community
The Steps 2 Success Community is where thousands of users help each other out and share ideas. Post a question or see if one has already been answered.

11:18 LTE

Before After

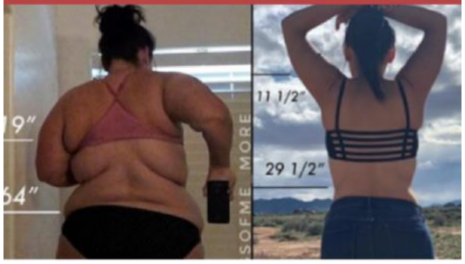

Inspiring Before and After Pictures

Change is hard, especially when results aren't immediate. Get motivated by real Step 2 Success users who have lost major pounds. These users have lost more than 50 pounds each. Be inspired by their weight loss success stories, and see their before and after pictures.

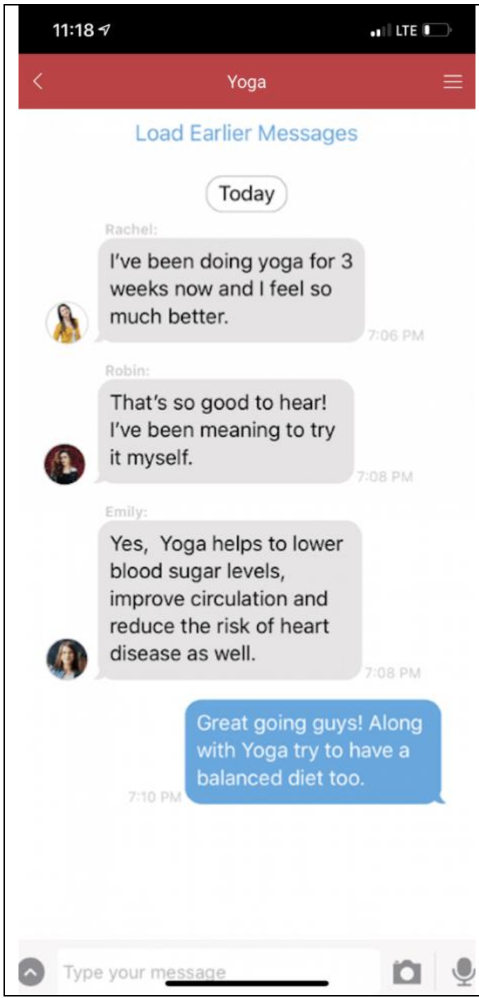
11:18
LTE
Yoga
Load Earlier Messages
Today
Rachel:
I've been doing yoga for 3 weeks now and I feel so much better.
7:06 PM
Robin:
That's so good to hear! I've been meaning to try it myself.
7:08 PM
Emily:
Yes, Yoga helps to lower blood sugar levels, improve circulation and reduce the risk of heart disease as well.
7:08 PM
Great going guys! Along with Yoga try to have a balanced diet too.
7:10 PM
Type your message

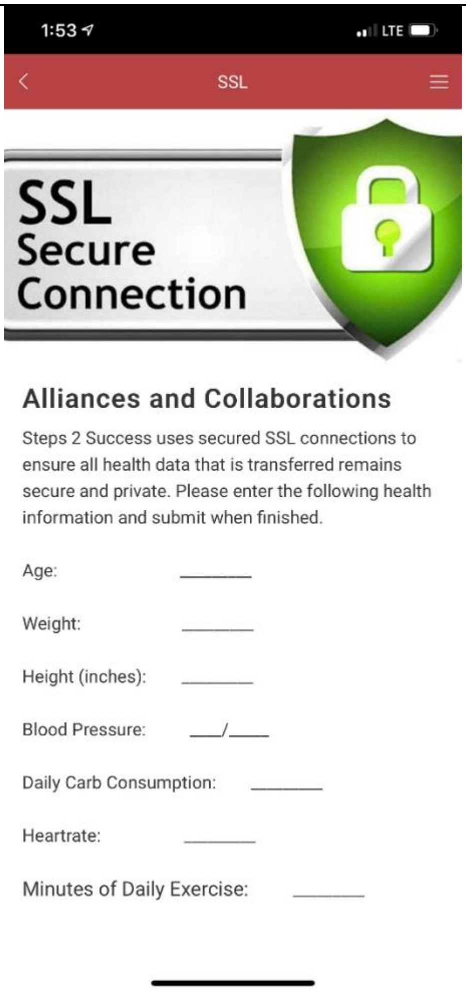
1:53
LTE
SSL
SSL
Secure
Connection
Alliances and Collaborations
Steps 2 Success uses secured SSL connections to ensure all health data that is transferred remains secure and private. Please enter the following health information and submit when finished.
Age: ______
Weight: ______
Height (inches): ______
Blood Pressure: ___/___
Daily Carb Consumption: ______
Heartrate: ______
Minutes of Daily Exercise: ______

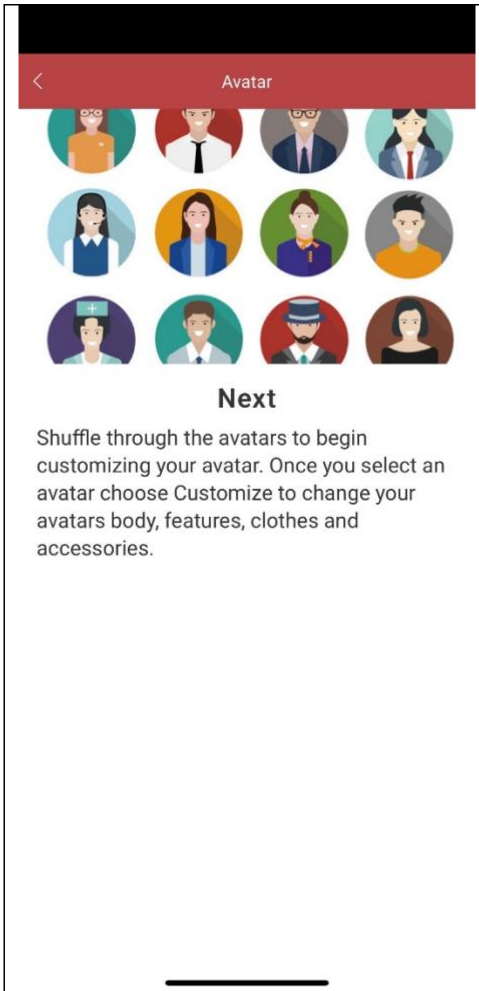
Avatar
Next
Shuffle through the avatars to begin customizing your avatar. Once you select an avatar choose Customize to change your avatars body, features, clothes and accessories.
