# Supplementary material for: The Intersection of Persuasive System Design and Personalization in Mobile Health: Statistical Evaluation
Source: JMIR Mhealth Uhealth. 2022 Sep 14;10(9):e40576. doi: 10.2196/40576 (PMC9520383; doi:10.2196/40576)
Supplement: Multimedia Appendix 2 [file mhealth_v10i9e40576_app2.docx]

**New General Self-Efficacy Scale**

Derived from the New General Self-Efficacy Scale

|  | Strongly disagree | Disagree | Somewhat disagree | Neither agree nor disagree | Somewhat agree | Agree | Strongly agree |
| --- | --- | --- | --- | --- | --- | --- | --- |
| I will be able to achieve most of the goals that I have set for myself. |  |  |  |  |  |  |  |
| When facing difficult tasks, I am certain that I will accomplish them. |  |  |  |  |  |  |  |
| In general, I think that I can obtain outcomes that are important to me. |  |  |  |  |  |  |  |
| I believe I can succeed at most any endeavor to  which I set my mind. |  |  |  |  |  |  |  |
| I will be able to successfully overcome many challenges. |  |  |  |  |  |  |  |
| I am confident that I can perform effectively on  many different tasks. |  |  |  |  |  |  |  |
| Compared to other people, I can do most tasks very well. |  |  |  |  |  |  |  |
| Even when things are tough, I can perform quite well. |  |  |  |  |  |  |  |
