# Supplementary material for: The Intersection of Persuasive System Design and Personalization in Mobile Health: Statistical Evaluation
Source: JMIR Mhealth Uhealth. 2022 Sep 14;10(9):e40576. doi: 10.2196/40576 (PMC9520383; doi:10.2196/40576)
Supplement: Multimedia Appendix 3 [file mhealth_v10i9e40576_app3.docx]

## Health Consciousness Scale

Derived from the Health Consciousness Scale

|  | Strongly disagree | Disagree | Somewhat disagree | Neither agree nor disagree | Somewhat agree | Agree | Strongly agree |
| --- | --- | --- | --- | --- | --- | --- | --- |
| I worry about the harmful  chemicals  in my food. |  |  |  |  |  |  |  |
| I am concerned  about my drinking  water quality. |  |  |  |  |  |  |  |
| I usually read the ingredients on  food labels. |  |  |  |  |  |  |  |
| I read more health related  articles than I did 3 years ago. |  |  |  |  |  |  |  |
| I am interested in information about my health. |  |  |  |  |  |  |  |
| I am concerned about my health all the  time. |  |  |  |  |  |  |  |
