# Supplementary material for: The Intersection of Persuasive System Design and Personalization in Mobile Health: Statistical Evaluation
Source: JMIR Mhealth Uhealth. 2022 Sep 14;10(9):e40576. doi: 10.2196/40576 (PMC9520383; doi:10.2196/40576)
Supplement: Multimedia Appendix 4 [file mhealth_v10i9e40576_app4.docx]

## Health Motivation Scale

Derived from the Health Motivation Scale

|  | Strongly disagree | Disagree | Somewhat disagree | Neither agree nor disagree | Somewhat agree | Agree | Strongly agree |
| --- | --- | --- | --- | --- | --- | --- | --- |
| I try to prevent common health problems before I feel any symptoms. |  |  |  |  |  |  |  |
| I am concerned about common health risks and try to take action to prevent them. |  |  |  |  |  |  |  |
| I don't worry about common health risks until they become a problem for me or someone close to me. |  |  |  |  |  |  |  |
| Because there are so many illnesses that can hurt me these days, I am not going to worry about them. |  |  |  |  |  |  |  |
| I don't take any action against common health risks I hear about until I know I have a problem. |  |  |  |  |  |  |  |
| I would rather enjoy life than try to make sure I am not exposing myself to health risks. |  |  |  |  |  |  |  |
