# Supplementary material for: The Intersection of Persuasive System Design and Personalization in Mobile Health: Statistical Evaluation
Source: JMIR Mhealth Uhealth. 2022 Sep 14;10(9):e40576. doi: 10.2196/40576 (PMC9520383; doi:10.2196/40576)
Supplement: Multimedia Appendix 5 [file mhealth_v10i9e40576_app5.docx]

**Big 5 Mini-IPIP Scale**

Derived from the Big 5 Mini-IPIP Scale

|  | Extremely Inaccurate | Moderately Inaccurate | Slightly Inaccurate | Neither Accurate nor Inaccurate | Slightly Accurate | Moderately Accurate | Extremely Accurate |
| --- | --- | --- | --- | --- | --- | --- | --- |
| Am the life of the party. |  |  |  |  |  |  |  |
| Sympathize with others'  feelings. |  |  |  |  |  |  |  |
| Get chores done right away. |  |  |  |  |  |  |  |
| Have frequent mood swings. |  |  |  |  |  |  |  |
| Have a vivid imagination. |  |  |  |  |  |  |  |
| Don’t talk a lot. |  |  |  |  |  |  |  |
| Am not interested in other people’s problems. |  |  |  |  |  |  |  |
| Often forget to put things back in their proper place. |  |  |  |  |  |  |  |
| Am relaxed most of the time. |  |  |  |  |  |  |  |
| Am not interested in abstract ideas. |  |  |  |  |  |  |  |
| Talk to a lot of different people at parties. |  |  |  |  |  |  |  |
| Feel others’ emotions. |  |  |  |  |  |  |  |
| Like order. |  |  |  |  |  |  |  |
| Get upset easily. |  |  |  |  |  |  |  |
| Have difficulty understanding abstract ideas. |  |  |  |  |  |  |  |
| Keep in the background. |  |  |  |  |  |  |  |
| Am not really interested in others. |  |  |  |  |  |  |  |
| Make a mess of things. |  |  |  |  |  |  |  |
| Seldom feel blue. |  |  |  |  |  |  |  |
| Do not have a good imagination. |  |  |  |  |  |  |  |
