# Supplementary material for: The Intersection of Persuasive System Design and Personalization in Mobile Health: Statistical Evaluation
Source: JMIR Mhealth Uhealth. 2022 Sep 14;10(9):e40576. doi: 10.2196/40576 (PMC9520383; doi:10.2196/40576)
Supplement: Multimedia Appendix 6 [file mhealth_v10i9e40576_app6.docx]

**Perceived Persuasiveness Scale**

Derived from the Perceived Persuasiveness Scale

|  | Strongly disagree | Disagree | Somewhat disagree | Undecided | Somewhat agree | Agree | Strongly agree |
| --- | --- | --- | --- | --- | --- | --- | --- |
| This mobile health screen has an influence on me. |  |  |  |  |  |  |  |
| This mobile health screen is personally relevant to me. |  |  |  |  |  |  |  |
| This mobile health screen makes me reconsider my overall health and wellness. |  |  |  |  |  |  |  |
