# Supplementary material for: The Intersection of Persuasive System Design and Personalization in Mobile Health: Statistical Evaluation
Source: JMIR Mhealth Uhealth. 2022 Sep 14;10(9):e40576. doi: 10.2196/40576 (PMC9520383; doi:10.2196/40576)
Supplement: Multimedia Appendix 7 [file mhealth_v10i9e40576_app7.pdf]

Weighted Perceived Persuasiveness (Male Under 40)	Weighted Perceived Persuasiveness (Female Under 40)
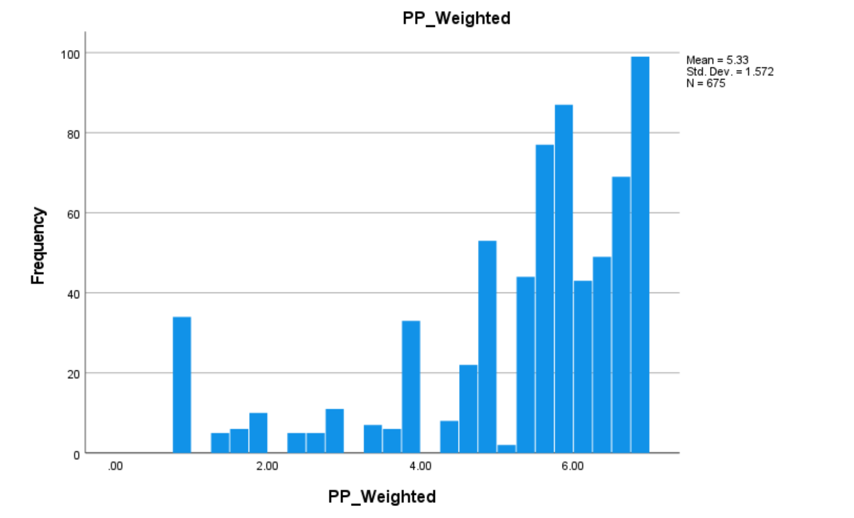	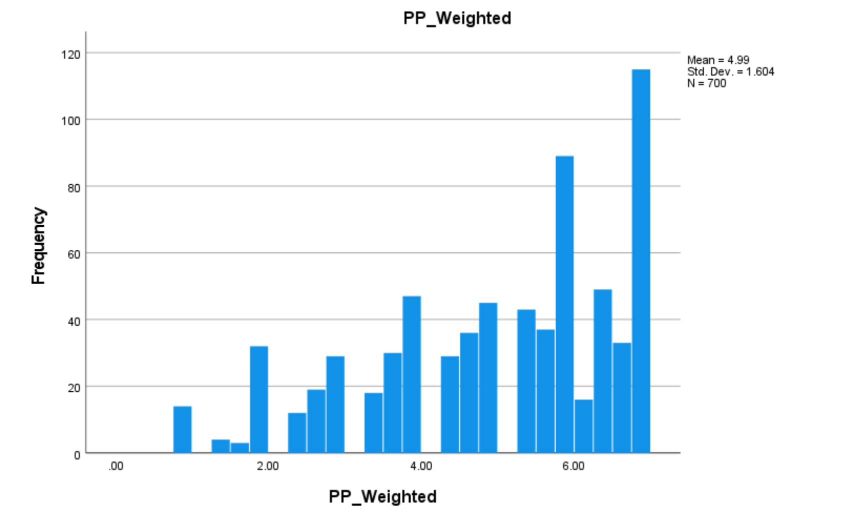

Weighted Perceived Persuasiveness
(Male 40-59)

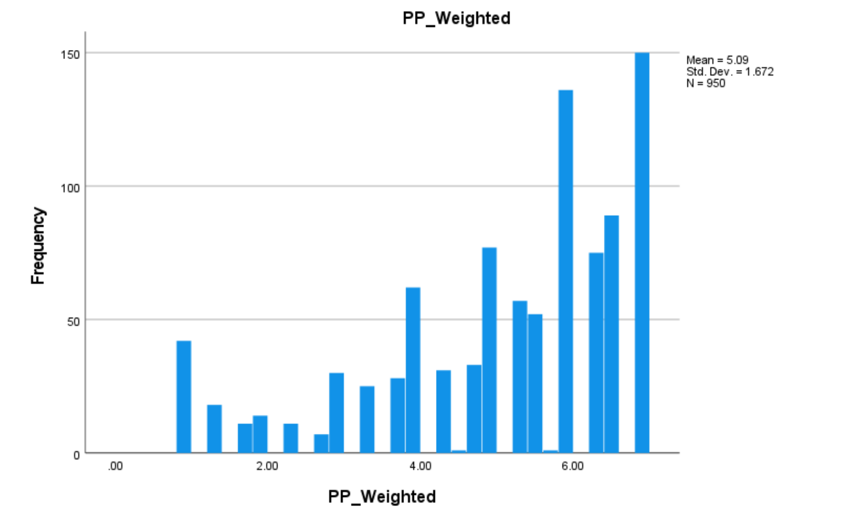

Weighted Perceived Persuasiveness
(Female 40-59)

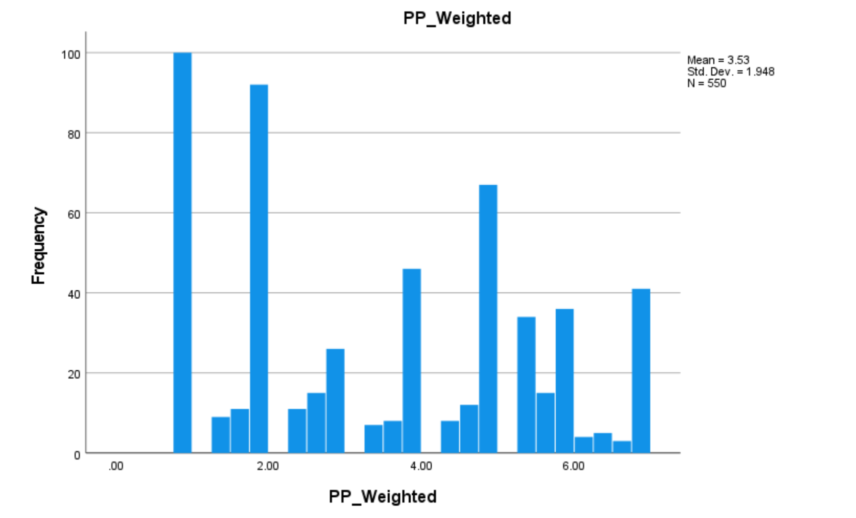

Weighted Perceived Persuasiveness (Male 60 Plus)	Weighted Perceived Persuasiveness (Female 60 Plus)
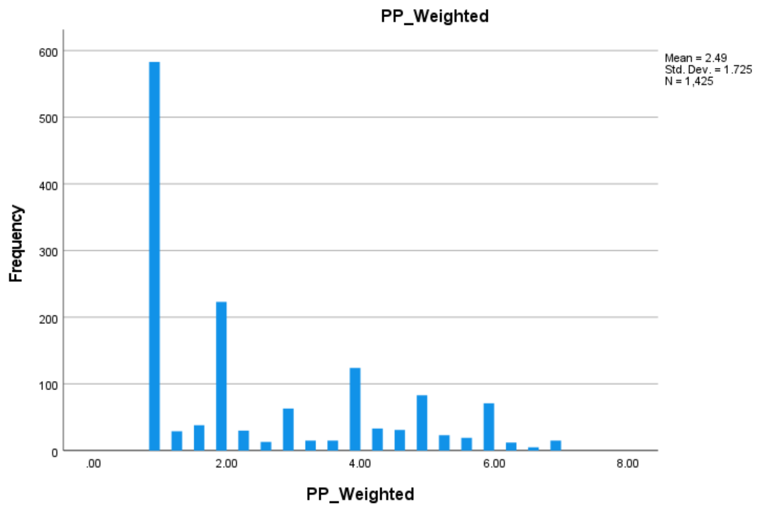	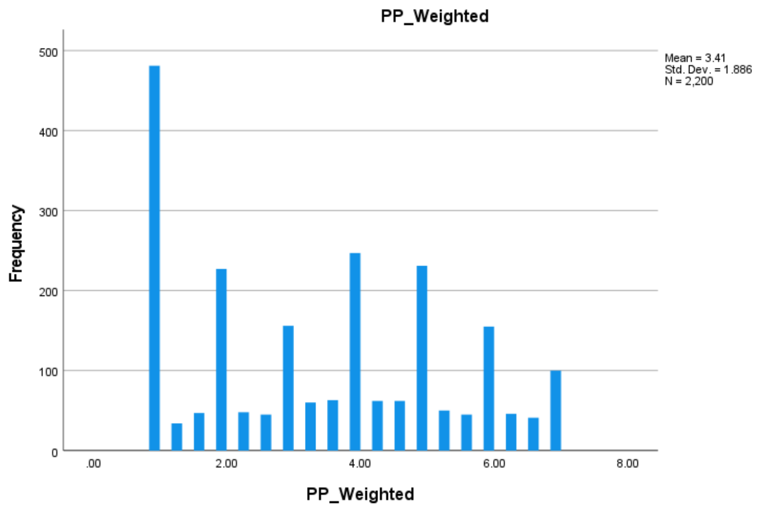
